# Inhaled sedation versus propofol in respiratory failure in the ICU (INSPiRE-ICU2): study protocol for a multicenter randomized controlled trial

**DOI:** 10.1186/s13063-025-08791-0

**Published:** 2025-03-31

**Authors:** Brian O’Gara, Alexis L. Serra, Joshua A. Englert, Alisha Sachdev, Robert L. Owens, Steven Y. Chang, Pauline K. Park, Daniel Talmor, Ida Sverud, Peter Sackey, Jeremy R. Beitler

**Affiliations:** 1 Department of Anesthesia, Critical Care, and Pain Medicine, Beth Israel Deaconess Medical Center and Harvard Medical School, Boston, MA USA; 2 ASPIRE Trials Program, Division of Pulmonary, Critical Care, and Sleep Medicine, New York University, 462 First Ave, New York, NY 10016 USA; 3 Division of Pulmonary, Critical Care, and Sleep Medicine, The Ohio State University Wexner Medical Center, Columbus, OH USA; 4 Department of Anesthesiology, Rush University Medical Center, Chicago, IL USA; 5 Division of Pulmonary, Critical Care, Sleep Medicine, and Physiology, University of California San Diego, San Diego, CA USA; 6 Department of Medicine, David Geffen School of Medicine, University of California Los Angeles, Los Angeles, CA USA; 7 Division of Acute Care Surgery, Department of Surgery, University of Michigan, Ann Arbor, MI USA; 8 Sedana Medical AB, Danderyd, Sweden; 9 Department of Physiology and Pharmacology, Karolinska Institutet, Stockholm, Sweden

**Keywords:** Inhalation anesthetic, Isoflurane, Propofol, Moderate sedation, Deep sedation, Respiratory failure, Randomized controlled trial, Clinical trial protocol

## Abstract

**Background:**

Patients undergoing invasive mechanical ventilation often require pharmacologic sedation to facilitate tolerance of this life-sustaining intervention, but sedatives currently used in routine care have substantial limitations. Isoflurane is an inhaled volatile anesthetic with pharmacologic properties potentially suitable to sedation of ventilator-dependent critically ill patients, but need for specialized drug administration equipment has limited its use historically to general anesthesia in the operating theatre. This trial will evaluate isoflurane, administered using a novel drug delivery system, for sedation of ventilator-dependent adult intensive care unit (ICU) patients in the United States (US).

**Methods:**

The Inhaled Sedation versus Propofol in Respiratory Failure in the ICU (INSPiRE-ICU2) is a phase 3, multicenter, randomized, controlled, assessor-blinded non-inferiority trial that will evaluate efficacy and safety of inhaled isoflurane delivered via the Sedaconda ACD-S, compared to intravenous propofol, for sedation of mechanically ventilated adult ICU patients. At 16 US hospitals, 235 enrolled patients requiring continuous sedation during invasive mechanical ventilation will be randomized in 1.5:1 ratio to inhaled isoflurane or intravenous propofol for sedation. Treatment duration is expected to be at least 12 h and may last up to 48 (± 6) h or until no longer needing continuous sedation, whichever occurs first. The primary endpoint is the percentage of time sedation depth is maintained within the targeted range (Richmond Agitation Sedation Scale − 1 to − 4), in the absence of rescue sedation, during the treatment period. Secondary superiority outcomes include opioid exposure, wake-up time, cognitive recovery after end-of-treatment, and preservation of spontaneous breathing effort.

**Discussion:**

The INSPiRE-ICU2 trial will help determine the potential role of isoflurane for sedation of ventilator-dependent adult patients in the ICU. Key trial design features, including adoption of the estimand framework and blinded assessments of sedation depth, pain, and cognitive recovery, will ensure a rigorous evaluation of isoflurane for ICU sedation.

**Trial registration:**

ClinicalTrials.gov, NCT05327296. First registered on April 5, 2022.

**Supplementary Information:**

The online version contains supplementary material available at 10.1186/s13063-025-08791-0.

## Background

An estimated 20–40% of intensive care unit (ICU) patients in the United States (US) receive invasive mechanical ventilation [1]. Most require sedation to facilitate tolerance of invasive respiratory support [2]. In US ICUs, sedation is most often achieved with intravenous propofol, dexmedetomidine, or midazolam, which have an established record of safety and efficacy but also clear downsides. Propofol, typically considered standard of care, facilitates faster wake-up than benzodiazepines [3], but hypotension is common, and propofol-related infusion syndrome (PRIS) can be life-threatening. Dexmedetomidine has limited utility for deeper sedation and can cause bradycardia and hypotension [4]. Benzodiazepines are lipophilic, some (e.g., midazolam) have active metabolites, and they may increase risk of delirium and delay wake-up upon discontinuation [5]. Opioid-based analgosedation often precipitates respiratory depression and constipation and can delay wake-up—particularly for those opioids that are metabolized in the liver or are renally cleared. Limitations of existing sedatives, coupled with recent sedative drug shortages [6], indicate a need for alternative sedatives for mechanically ventilated patients.

Isoflurane, an inhaled volatile anesthetic, has been used routinely in operating rooms throughout the world for general anesthesia for over half a century. Several properties of isoflurane make it potentially appealing for ICU sedation. Isoflurane has favorable pharmacokinetics and pharmacodynamics, including rapid uptake, rapid elimination, and negligible accumulation in adipose tissue. These pharmacologic properties translate not only to rapid onset of sedation but also faster wake-up when discontinued compared to propofol [7]. Furthermore, isoflurane is eliminated, unmetabolized almost exclusively by the lungs, and unaffected by renal, hepatic, or multiorgan dysfunction that is commonplace in ICU patients. Inhalational drug delivery is effective even in severe lung injury, such as from ARDS [8]. Like some other sedatives (e.g., propofol), dose-dependent vasodilatory hypotension is the most common side-effect, but its potential advantages warrant consideration for ICU sedation.

Historically, administration of inhaled anesthetic has required an anesthesia machine not designed for supporting ICU patients with severe respiratory failure [9]. Given this need for specialized equipment, clinical experience with volatile anesthetics outside the operating theater has been limited to rare cases such as status asthmaticus and status epilepticus [10].

The Anesthetic Conserving Device (ACD, Sedana Medical, Daneryd, Sweden) is designed to administer volatile anesthetics on an ICU ventilator. Isoflurane via the ACD was evaluated in an unblinded phase 3 trial comparing it to propofol for up to 54 h of sedation of invasively ventilated adult patients [7]. Isoflurane was non-inferior to propofol for maintaining targeted sedation depth and was associated with significantly faster wake-up times and decreased opioid exposure. However, the trial was unblinded and conducted in German and Slovenian ICUs with some prior clinical experience using volatile anesthetics via the ACD for ICU sedation. Other studies have similarly suggested that inhaled anesthetic sedation via the ACD may lead to faster wake-up times, earlier extubation, and decreased opioid use compared to intravenous sedatives [11–15]. Isoflurane sedation via the ACD is currently approved for clinical use in ICUs in 18 European countries, and the ACD is approved in several Latin American and Asian countries, but neither the ACD nor administration of isoflurane for sedation in the ICU is currently approved for clinical use in the US.

INSPiRE-ICU2 (NCT05327296) is a phase 3, multicenter, randomized, controlled, assessor-blinded non-inferiority trial to evaluate efficacy and safety of inhaled isoflurane delivered via the Sedaconda ACD-S compared to intravenous propofol for sedation of mechanically ventilated adult ICU patients in the US. The primary hypothesis is that isoflurane administered via the ACD is non-inferior to propofol in sedation efficacy, as defined by time spent within target Richmond Agitation Sedation Scale (RASS) goal. Key secondary endpoints include concomitant opioid use, wake-up time, cognitive recovery after end-of-treatment, and preservation of spontaneous breathing effort.

## Methods

### Participants

A total of 235 randomized participants will be enrolled from 16 hospitals in the US. Each site will obtain Institutional Review Board (IRB) approval to conduct the study. Ethical approval will be obtained from a central IRB (Advarra, Columbia, MD) where permitted by local site regulations. In instances where local IRB policy does not allow for ceded review, the site’s own local IRB will remain the IRB of record for the study.

Eligible are adult ICU patients undergoing invasive mechanical ventilation who are expected to have clinical need for continuous sedation to achieve goal RASS − 1 to − 4 for > 12 h. Exclusion criteria center on potential contraindications to the ACD, isoflurane, or propofol, clinical need for other sedatives, neurological conditions that preclude study procedures, and mechanical ventilation for > 72 h prior to enrollment (Table 1). Written informed consent will be obtained by the site investigators or designated study personnel, as documented on site responsibility logs, from each participant or their legally authorized representative. Patients scheduled for surgery with anticipated need for postoperative ventilation > 12 h may be approached preoperatively for consent but can only be enrolled/randomized once eligibility criteria are confirmed postoperatively. Eligible patients otherwise will be identified via daily screening of ICU patient lists.

**Table 1:** Eligibility criteria for INSPiRE-ICU2

**Inclusion Criteria**
1. Adults ≥ 18 years of age
2. Patients who are anticipated to require > 12 h of invasive mechanical ventilation and continuous sedation in the ICU
3. Receipt of continuous sedation due to clinical need for sedation to RASS < 0
**Exclusion Criteria**
1. Need for RASS − 5
2. Sedation for invasive mechanical ventilation immediately prior to Baseline for > 72 h (patients who have been extubated for at least 24 h and subsequently re-intubated will have sedation for invasive mechanical ventilation starting from when they were re-intubated)
3. Severe neurological condition that causes the patient to lack ability to participate in the study (i.e., unable to be assessed for RASS and Critical Care Pain Observation Tool), including, but not restricted to, patients with acute stroke, severe head trauma, meningitis, suspected of having elevated intracranial pressure (ICP), or the need for ICP monitoring
4. Ventilator tidal volume < 200 or > 1000 mL at baseline
5. Need for extracorporeal membrane oxygenation (ECMO), extracorporeal CO_2_ removal (ECCO2R), high frequency oscillation ventilation (HFOV), or high-frequency percussive ventilation (HFPV) at screening
6. Comfort care only (end of life care)
7. Contraindication to propofol or isoflurane, including: a. Known or suspected personal or family history of malignant hyperthermia (MH) or high risk for MH or acute drug-induced muscle injury (e.g., muscular dystrophies); b. Severe hemodynamic compromise, defined as the need for norepinephrine ≥ 0.3 mcg/kg/min (or equivalent vasopressor dose) to maintain blood pressure within acceptable range, assumed to be mean arterial pressure ≥ 65 mmHg unless prescribed clinically; or c. Allergy to isoflurane or propofol, or have propofol infusion syndrome
8. History of ventricular tachycardia/long QT syndrome
9. Requirement of IV benzodiazepine or barbiturate administration for seizures or dependencies, including alcohol withdrawal
10. Neuromuscular disease that impairs spontaneous ventilation (e.g., C5 or higher spinal cord injury, amyotrophic lateral sclerosis)
11. Concurrent enrollment in another study that, in the Investigator’s opinion, would impact the patient’s safety or assessments of this study
12. Participation in other study involving investigational drug(s) or devices(s) within 30 days prior to randomization
13. Previous randomization or receipt of treatment in this study or in INSPiRE-ICU1;
14. Anticipated requirement of treatment with continuous infusion of a neuromuscular blocking agent for > 4 h
15. Female patients who are pregnant or breast-feeding
16. Imperative need for continuous active humidification through ventilator circuit
17. Attending physician’s refusal to include the patient
18. Inability to obtain informed consent

### Training

During site activation, study personnel will receive in-person training from respiratory therapist Clinical Education Specialists. Training topics include isoflurane administration, the ACD and ventilator circuit, blinding procedures, and standardization of study assessments. Sites will also identify clinical staff to serve as “super users” who will receive training to provide peer support for clinical teams involved in the care of enrolled participants. In-person training will be supplemented by online modules, videos, quick guides, and competency lists specific to clinical roles to accommodate different learning styles. The Clinical Education Specialists will be available for the duration of the trial to provide guidance and support for research and clinical staff.

### Run-in phase

Before opening to randomization, each site must enroll at least 1—and up to 5—non-randomized patient(s) who will receive isoflurane. This run-in phase will promote site familiarity with the Sedaconda ACD-S, isoflurane titration, and other study procedures. During the run-in phase, all trial procedures will be conducted according to protocol except that blinded assessments are not required. Run-in participants are excluded from efficacy analyses.

### Randomization

Participants will be enrolled and randomized by site investigators or designated study personnel via a central web-based system to inhaled isoflurane or intravenous propofol in a 1.5:1 ratio. Weighted randomization will increase data available on isoflurane via the ACD for a new clinical indication. The allocation sequence will remain concealed from site investigators for the duration of the trial and will be centrally managed and implemented via the use of an interactive response technology system that is only accessed once a participant is enrolled. Randomization will be stratified by type of admission (medical/surgical) and Simplified Acute Physiology Score (SAPS) III score (< 40, 40 to 59, or ≥ 60) to ensure balance across these key patient subgroups.

### Isoflurane administration system

**Fig. 1:**
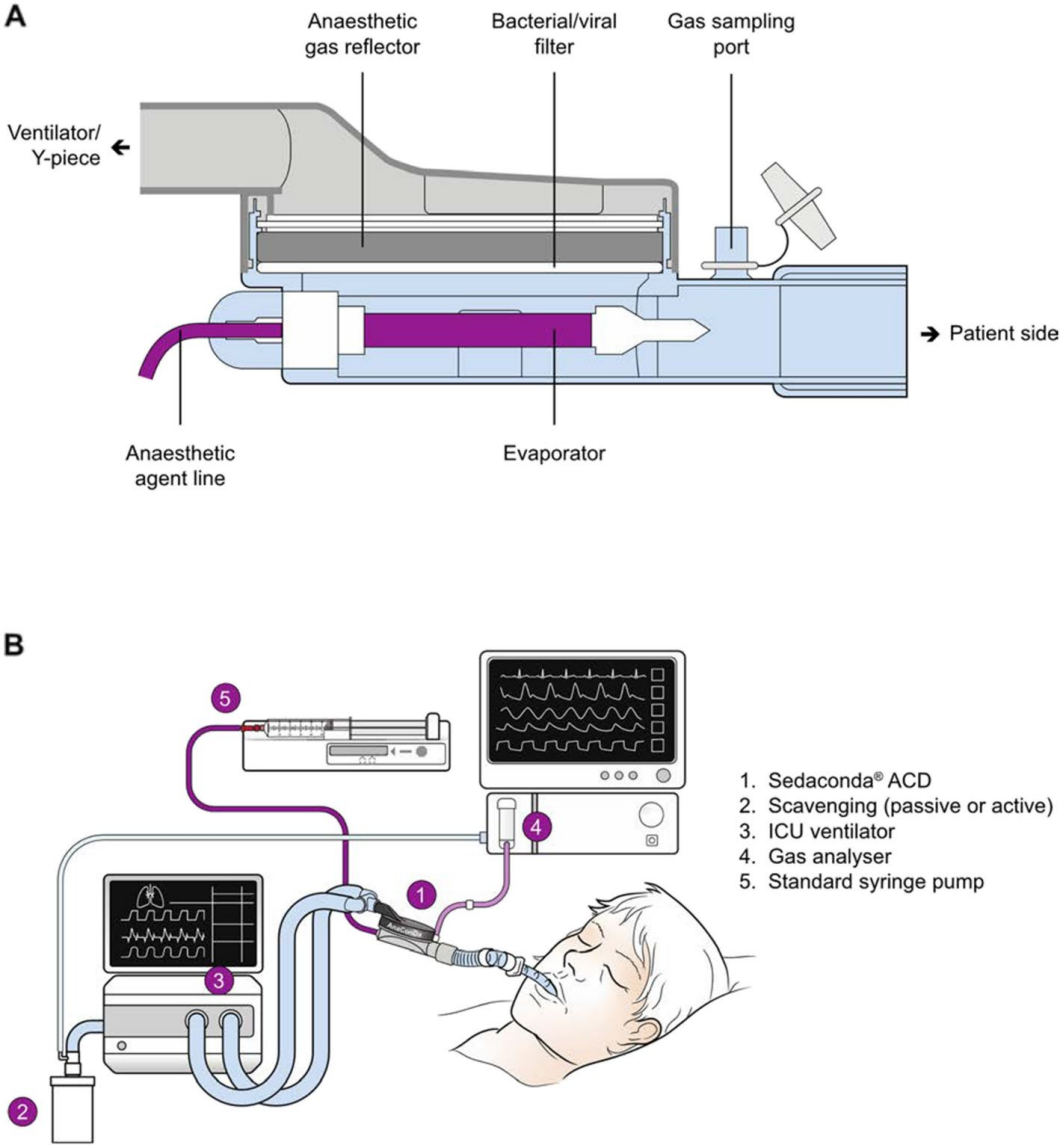
Sedaconda ACD schematic and ventilator circuit adaptations. **A** Cross-sectional internal schematic view of the Sedaconda ACD, which can be thought of as a heat-moisture exchanger (HME) modified to facilitate efficient administration of volatile anesthetics. Two key adaptations to facilitate drug delivery are the infusion line/evaporator rod through which drug enters the ventilator circuit, and the anesthetic gas reflector that adsorbs 90% of exhaled anesthetic, which is then re-breathed with the following inspiratory cycle. **B** Configuration of the ventilator circuit for administration of volatile anesthetic via the Sedaconda ACD. Though the gas analyzer can be used to monitor end-tidal concentration of isoflurane, the drug is titrated not to any particular concentration but rather to clinically ascertained sedation depth using Richmond Agitation Sedation Scale (RASS)

Administration of isoflurane on the ICU ventilator circuit requires a few adaptations (Fig. 1). The ACD is a specialized heat and moisture exchanger (HME) that connects between the endotracheal tube and ventilator Y-piece, precluding use of active humidification. The ACD includes a perforated vaporizing rod for anesthetic delivery and carbon filter for anesthetic rebreathing. Volatile anesthetic infuses into the ACD’s vaporizing rod via a syringe pump, where it evaporates and is inhaled by the patient during the inspiratory phase. During exhalation, the ACD’s carbon filter adsorbs exhaled anesthetic, preventing approximately 90% of the delivered medication from entering the expiratory limb of the ventilator circuit [16]. With the next ventilator cycle, inspiratory air flow frees the adsorbed anesthetic, which is inhaled again by the patient, effectively resulting in anesthetic rebreathing. As with any HME, the ACD adds a modest volume of dead-space to the circuit. The ACD-S, used in INSPiRE-ICU2, adds approximately 50 mL of dead-space volume to the circuit.

In addition to the ACD, a scavenger is connected to the ventilator exhaust to prevent ambient release of the roughly 10% of drug that enters the expiratory limb with each cycle. A sampling line is connected to measure end-tidal concentration of anesthetic gas.

### Blinding

Due to clinical need for titratable sedative, a double-blind placebo-controlled trial was deemed infeasible. Bedside clinical staff directly involved in the participant’s care will be informed of treatment assignment as needed to titrate study drug and analgesia, guided by their bedside assessment of sedation and pain using RASS and CPOT, respectively. Otherwise, treatment allocation will not be disclosed unless clinically necessary. Key study endpoints—including those incorporating assessment of sedation depth, pain, and cognitive recovery—will be ascertained independently by blinded assessors uninvolved in the participant’s care and without knowledge of treatment group.

**Fig. 2:**
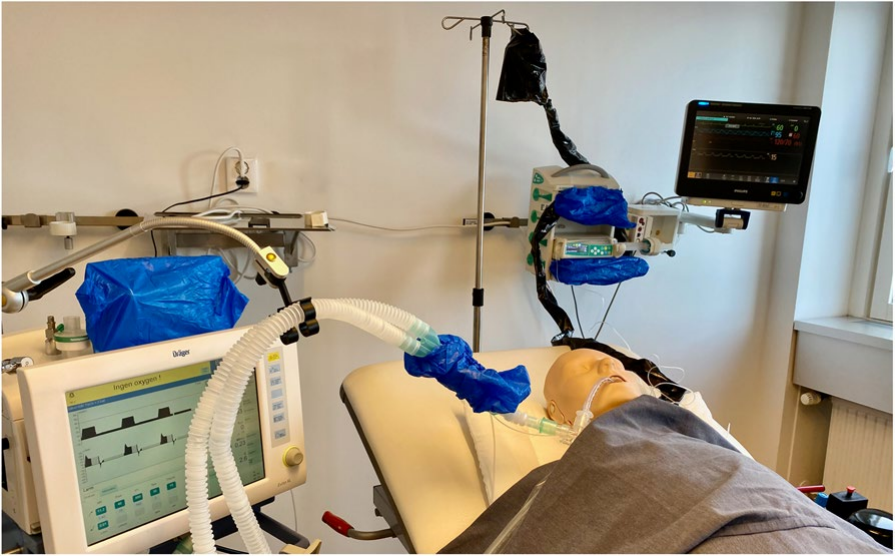
Blinding set-up. Blinding study drug entails unique considerations because propofol is a white intravenous infusion while isoflurane is a clear colorless volatile liquid that is administered via a specialized inhalational delivery device, the Sedaconda ACD. Plastic wraps are used to cover the propofol bottle and infusion line (or their dummy equivalent), as well as the Sedaconda ACD (or heat-moisture exchanger) placed inline with the ventilator circuit. A volatile anesthetic scavenger is placed on the ventilator exhaust and gas sampling monitor connected to the ACD or heat-moisture exchanger in both arms (not shown). The gas sampling monitor displays only end-tidal carbon dioxide by default to preserve blinding, though the study team can access additional data views as needed for periodic recording of end-tidal isoflurane concentration in patients assigned to the intervention arm

To facilitate blinding, two study medication delivery systems will be placed in the patient room: a functional setup for active treatment and a nonfunctional sham setup. The ventilator circuit will utilize passive humidification, with either the ACD (isoflurane arm) or a similar-sized HME (propofol arm) for heating and humidification of inspired air. Opaque covers will be placed over the ACD or HME to blind the ventilator circuit, over a syringe pump and IV infusion pump, and over the propofol bottle and IV tubing (or sham equivalent) to blind the infusion setup (Fig. 2).

### Interventions

As soon as possible after randomization and prior to study drug initiation, standard of care sedative and opioid infusion rates will be halved to minimize their effects during the study drug treatment period. If needed, they can be titrated further prior to the start of study medication.

**Fig. 3:**
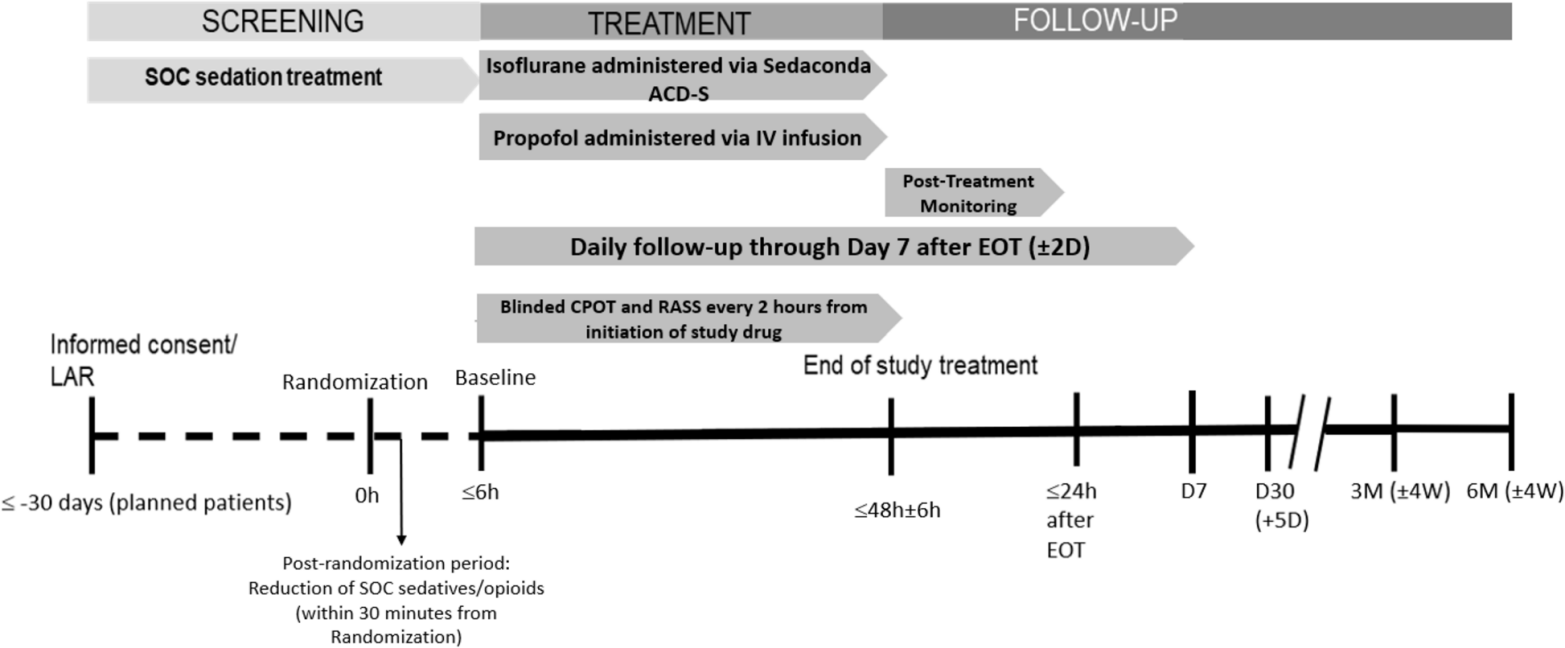
Study scheme. Key events during the screening, post-randomization, treatment, and follow-up periods are depicted. *Abbreviations*: CPOT = Critical Care Pain Observation Tool; D = day(s); EOT = end of treatment; h = hour(s); IV = intravenous; LAR = legally authorized representative; M = month; RASS = Richmond Agitation Sedation Scale; Sedaconda ACD-S = Sedaconda Anaesthetic Conserving Device - S; SOC = standard of care; W = week(s)

Study drug (isoflurane or propofol) will be initiated within 6 h after randomization (Fig. 3). Standard of care sedatives will be discontinued at the time of study drug initiation.

For patients assigned to the intervention arm, inhaled isoflurane will be initiated at a dose of up to 3.0 mL/h, with starting dose < 3.0 mL/h recommended in deeply sedated or hypotensive patients requiring vasopressors. Thereafter, isoflurane may be titrated every 10–15 min in increments of 0.5–1.0 mL/h, up to a maximum rate of 15 mL/h, as needed to achieve target sedation depth as determined by the clinical team. Bolus doses of 0.3–0.5 mL may be administered for rapid deepening of sedation if needed.

For patients assigned to the control arm, intravenous propofol will be initiated at 0.6–1.5 mg/kg/h, or at the pre-randomization dose if applicable and within the target RASS range. Propofol may be adjusted every 5–10 min in increments of 0.3–0.6 mg/kg/h, up to a maximum rate of 4.0 mg/kg/h, until the sedation target is attained. Bolus doses of 0.3–0.5 mg/kg may be administered for rapid deepening of sedation.

For both arms, study drug will be titrated as needed to maintain target RASS between − 1 and − 4 during the treatment period. Study drug and rescue sedation will not be used to treat pain. Opioids and other analgesic medications (e.g., acetaminophen, ketorolac, ibuprofen) are permitted throughout the study to treat pain. All study patients should receive adequate analgesia throughout the study at the discretion of the investigator and treating clinical team. Continuous opioid infusions as per standard of care are allowed throughout the study drug treatment period and may be adjusted as clinically appropriate (i.e., guided by CPOT). The lowest possible opioid maintenance dose to reach analgesia and comfort goals and minimize opioid side effects should be used.

When study drug is insufficient to maintain targeted sedation depth, dexmedetomidine 0.15–0.7 mcg/kg/h for up to 3 h/day or midazolam 0.5–5 mg per bolus for up to 3 boluses/day can be used as rescue. Need for rescue sedation beyond these thresholds will be deemed treatment failure. Other sedatives, including barbiturates, clonidine, and ketamine, are prohibited during the study treatment period. Neuroleptics (e.g., haloperidol, quetiapine) should not be used during the study treatment period unless the participant was receiving these agents prior to ICU admission. Continuous neuromuscular blockade > 4 h is not permitted during the study treatment period. Temporary deepening of sedation (e.g., for minor ICU procedures) or lightening of sedation (e.g., for neurological assessment or daily spontaneous awakening test) is permitted, consistent with standard of care.

Treatment duration is expected to be at least 12 h and will continue for up to 48 h (± 6 h) or until there is no longer clinical need for continuous sedation, whichever occurs first. Study medication also should be discontinued for treatment failure, clinical need for a prohibited medication, new-onset coma due to structural brain disease, treatment-related severe or serious adverse events, two severe or any serious adverse event without a clear alternate explanation, other adverse events or medical condition whereupon discontinuation is deemed in the patient’s best interest, need for extracorporeal life support or a prohibited ventilator mode (high-frequency oscillatory or percussive ventilation), transition to comfort care, or withdrawal of consent.

### End-of-treatment and follow-up periods

Unless contraindicated, a wake-up test will be conducted at end-of-treatment to capture the time elapsed from sedative/opioid discontinuation until the participant is fully awake (RASS ≥ 0). Blinded RASS will be assessed every 15 min for the first hour after end-of-treatment and every 30 min thereafter until 4 h have elapsed or RASS ≥ 0 is attained. If needed, standard of care sedation may then be initiated. One hour after end-of-treatment, a blinded Confusion Assessment Method (CAM)-ICU-7 will be done in patients not re-sedated with propofol or benzodiazepines to ascertain short-term cognitive recovery [17].

Participants will be followed by the local site for 30 days for clinical endpoints, during which time they will receive usual care at the clinical team’s discretion. A subset of participants will undergo additional remote cognitive assessments via telephone or video contact, conducted centrally at 3 and 6 months. Tables 2, and 3 detail the complete schedule of events.

**Table 2:** Schedule of procedures through treatment period

	Screening Period				Treatment Period				
	Screening/Randomization			Baseline					
Hour, Day, or Month (± Window)	Initial Screening D -30 to Randomization	Complete Screening-24h to Randomization	Randomization	Randomization to Initiation of Study Drug (up to 6 hours)	Q2h (±0.5h)	Q4h^nn^ (±0.5h)	Q8h (±2h)	Daily	EOT/Up to 48h (±6h)
Informed consent^d^	X								
Inclusion/exclusion criteria	X	X^e^							
Demographic information	X								
Medical/surgical history	X	X^e^							
Prior/concomitant relevant medications^f^		X		X	X				
Weight and height^g^		X							
Physical examination^h^		X							X
Clinical laboratory assessments^i^		X						X	
Pregnancy test^l^		X							
Physical function and outcomes^m^	X^kk^								
Cognitive function and outcomes^n^	X^kk^								
Reduction of SOC sedation and opioids to half				X^o^					
Discontinuation of SOC sedatives and opioids				X^ll^					
Patient characteristics^p^		X							
Predicted time remaining on ventilator^q^		X							
Ventilator parameters^r^				X			X^s^		
Blood gases^t^				X			X^s^		
Organ function (SOFA)^u^				X				X	
Randomization^v^			X						
Vital signs^w^				X^x^			X^s^		
RASS				X^x^	X^y^			X^z^	X^y^
CPOT				X^x^	X^y^				
Study drug administration^v^				X	X				
Isoflurane end-tidal concentration measurement^aa^						X			
Sedaconda ACD-S device deficiencies					X				
Adverse events^cc^					X				
Restraints								X	
CAM-ICU-7^ee^								X^z^	X^ee^
Wake-up test^ff^									X

*ABG* arterial blood gas, *AE* adverse event, *BPI* Brief Pain Inventory, *CAM-ICU-7* 7-point scale of the Confusion Assessment Method for the Intensive Care Unit, *CPOT* Critical Care Pain Observation Tool, *D* day, *ECLS* extracorporeal life support, *Ecrf* electronic case report form, *EOT* end of treatment, *EtCO*_2_ end-tidal carbon dioxide, *EW* early withdrawal, *FAQ* functional activities questionnaire, *FiO*_2_ fraction of inspired oxygen, *h* hour, *ICU* intensive care unit, *IES‑R* impact of event scale, *IQCODE* Informant Questionnaire on Cognitive Decline in the Elderly, *Katz ADL* Katz Index of Independence in Activity of Daily Living, *LAR* legally authorized representative, *LTO* Long-Term Outcomes, *M* month, *P0.1* airway occlusion pressure, *PC* pressure assist/control, *PEEP* positive end‑expiratory pressure, *PIP* peak inspiratory pressure, *PROMIS* Patient-Reported Outcomes Measurement Information System, *PS* pressure support, *Q* every, *RASS* Richmond Agitation Sedation Scale, *SAPS* Simplified Acute Physiology Score, *Sedaconda ACD‑S* Sedaconda Anesthetic Conserving Device – S, *SOC* standard of care, *SOFA* sequential organ failure assessment, *SpO*_2_ peripheral capillary oxygen saturation, *TICS* Telephone Interview for Cognitive Status, *UNS* unscheduled, *W* week(s), *WAIS* Wechsler adult intelligence scale, *WHODAS 2.0* World Health Organization Disability Assessment Schedule 2.0, *WMS* Wechsler memory scale

^a^Major ICU interventions, information on ICU care, and relevant concomitant medications are to be collected at Study Day 30 or at ICU discharge, whichever comes first. Level of care (can be collected retrospectively for the whole study period), duration of mechanical ventilation, late onset drug-induced liver injury, mortality, and follow-up of any unresolved AEs are to be performed through telephone call if not possible to retrieve information through medical records at Study Day 30 (regardless of ICU discharge status)

^b^Patients will be followed-up via telephone call at 3 and 6 months (±4 weeks)

^c^Unless consent is withdrawn, the patient should continue in the study for assessment and follow-up even if study drug is discontinued. If consent is withdrawn, patients will be encouraged to complete the EW visit, if possible

^d^Informed consent must be obtained from patient or patient’s LAR before any study-related procedures are performed. If the patient is unable to consent at the time of Screening, informed consent may be obtained from the patient’s LAR; however, information about the study will be given to, and consent obtained from, the patient as soon as the patient’s condition allows

^e^To be assessed only if Initial Screening is performed more than 24 hours prior to the Complete Screening

^f^All relevant prior and concomitant medications are recorded from ICU admission or 24 hours prior to initiation of study drug treatment, whichever is shortest, until the end of the 24-hour post‑treatment monitoring period. After this, only receipt of specific sedative, antipsychotic, and analgesic medications and relevant concomitant medications associated with ongoing AEs will be collected from 24 hours after EOT until Study Day 30 or ICU discharge, whichever comes first. All administered opioids, including the mean opioid dose assessment at 60 minutes prior to randomization, are to be collected also during Screening

^g^Body weight (lb) and height (inch) should be measured when possible or be estimated. Weight and height available in patient’s records can be used if measured within the last 7 days

^h^Physical examinations should be performed appropriately, per the patient’s condition. Any findings should be recorded on the physical examination eCRF

^i^Includes clinical chemistry, lipid profile, hematology, and coagulation. Screening/Baseline clinical laboratory tests, other than those pertaining to trial eligibility, may be collected at any time during the Complete Screening (-24 hours to 0 hour) or post-randomization period (0 hour to +6 hours) prior to initiation of study drug treatment

^j^Assessment should be performed once during post-treatment monitoring period, if the patient is still in the ICU. Analyses performed per SOC with an 18-to-48-hour window after EOT can be used

^k^Only applicable if EW is during study drug treatment period

^l^Female patients of childbearing potential must have a negative (serum or urine) pregnancy test prior to randomization. A pregnancy test obtained previously as part of usual care during this hospital episode that is documented in the patient’s medical record will suffice. Female patients not of childbearing potential are defined as female patients who have been postmenopausal for at least 1 year, have been surgically sterilized, or are 60 years of age

^m^Physical outcomes assess activities of daily living by the Katz ADL and Pfeffer FAQ

^n^Cognitive baseline will be assessed by the IQCODE. LTO will be assessed by TICS, WAIS IV-Digit Span, Hayling Sentence Completion Test, Controlled Oral Word Association, WMS-IV – Immediate Memory (Adult/Older Adult), WMS-IV – Delayed Memory (Adult/Older Adult), and PROMIS Cognitive Function questionnaire

^o^To be performed within 30 minutes from randomization

^p^SAPS III, reasons for ICU admission, ICU diagnosis criteria, hospital and ICU admission, time for intubation, and exposure of volatile anesthetics in the past 24 hours (and if yes, what drug [sevoflurane/isoflurane/desflurane]) to be assessed at Complete Screening

^q^Predicted remaining time on the ventilator for each patient to be collected just shortly before randomization in order to monitor the proportion of patients with longer (>24 hours) versus shorter (12 to 24 hours) exposure times

^r^Ventilator parameters include ventilator mode, set tidal volume, observed tidal volume, set rate, observed rate, observed minute volume, set PEEP, PS above PEEP, PC above PEEP, PIP, plateau pressure (once daily only), mean airway pressure, FiO_2_, SpO_2_, EtCO_2_, ventilator trigger, P0.1, and ABG

^s^These assessments will be performed more frequently when clinically indicated (as patients will often be observed continuously or more frequently than every 8 hours in clinical practice)

^t^Only applicable when arterial line is available

^u^Organ function will be assessed by SOFA once daily at Baseline, during the study drug treatment period, the 24-hour post-treatment period, and until 7 days after EOT

^v^After randomization, the study equipment will be set-up and study drug treatment shall be initiated as close to randomization as possible and no later than 6 hours after randomization

^w^Vital signs include systolic, diastolic, and mean arterial blood pressure, heart rate, SpO_2_, (measured by pulse oximetry; will not be assessed while patients are on ventilator support, as it will be captured as a ventilator parameter), respiratory rate (will not be recorded as part of vital sign assessments while patient is on ventilator support, as it will be captured in the ventilator parameter records as observed breathing rate), and body temperature

^x^Unblinded baseline assessment for RASS and CPOT should be performed within 30 minutes prior to initiation of study drug administration. Vital signs should be performed within 60 minutes prior to initiation of study drug administration

^y^Assessment will be performed in a blinded manner by a blinded assessor

^z^CAM-ICU-7 and RASS will be performed daily (at a minimum) during the study drug treatment period and until 7 days after EOT or until hospital discharge, whichever comes first. However, more frequent assessments will be performed when clinically indicated. RASS

^aa^A separate gas monitor will be readily available during the study drug treatment period for measurement of end-tidal isoflurane concentrations. Only applicable for isoflurane‑treated patients

^bb^Major ICU interventions through Study Day 30 or until ICU discharge, whichever comes first, include the following: renal replacement therapy, ECLS, tracheostomy, non‑invasive ventilation, and re-admission to the ICU

^cc^Recording of AEs will start at the initiation of study drug administration and continue until Day 7 post EOT

^dd^Only AEs unresolved at D7 to be followed-u

^ee^CAM-ICU-7 will be assessed 60 (±10) minutes after EOT in all patients by a blinded assessor. CAM-ICU-7 will not be required for patients reaching EOT due to treatment failure, patients transitioned to comfort care, or patients continued onto benzodiazepines or propofol sedation due to clinical need before 60 minutes after EOT

^ff^Wake-up test will be assessed through blinded RASS assessments

^gg^To be collected for patients who are extubated on study drug only

^hh^Only applicable if EW is after EOT

^ii^Memory panorama from time in the ICU will be assessed by the ICU Memory Tool at the 3-month follow-up visit only

^jj^Psychological outcomes will be assessed by the PROMIS Depression and Anxiety questionnaires and IES-R

^kk^Quality of life will be assessed by the WHODAS 2.0 and BPI questionnaires

^ll^Baseline data can be collected until 48 hours after initiation of study drug treatment as the time period of interest is not synonymous with when data needs to be captured

^mm^As soon as the study drug treatment has been initiated, the SOC sedative should be stopped (ie, slow weaning will not be permitted) to limit the impact of residual SOC sedation

^nn^For isoflurane-treated patients only

^oo^LTO assessments will be completed at 3 and 6 months. ICU Memory Tool will be assessed at 3 months follow-up only

### Primary outcome

The primary outcome is the percentage of time sedation depth is maintained within the targeted range (RASS − 1 to − 4), in the absence of rescue sedation, during the treatment period. Sedation depth (RASS) will be measured by trained blinded assessors with no knowledge of treatment allocation. RASS assessments performed by unblinded clinical staff for titrating sedatives and opioids will not be used. Blinded assessments are scheduled to be performed every 2 (± 0.5) h throughout the study treatment period. Each scheduled assessment will be coded as “Success,” “Failure,” or “Censored.” “Success” is defined by sedation depth within the target range. “Failure” includes sedation depth outside the target range and the following intercurrent events: need for rescue sedation, treatment discontinuation for lack of efficacy, or death causally related to study drug. The maximum failure time to be assigned around an out of sedation depth range is ± 60 min. “Censored” time is defined by select intercurrent events (Table 4) and omitted in calculating the primary outcome. The number of minutes that each discrete RASS assessment deemed “Success” contributes to the primary endpoint depends on neighboring RASS assessments and intercurrent events (Table 4). The primary outcome is calculated as follows:

**Table 3:** Schedule of procedures during post-treatment follow-up period

	Post-Treatment Monitoring	Follow‑Up Contact		3-Month Phone Call	6‑Month Phone Call	EW^c^
Hour, Day, or Month (± Window)	Until 24h after EOT	Until D7 after EOT (±2D)	D30 (+5D)^a^	3M (±4W)^b^	6M (±4W)^b^	UNS
Prior/concomitant relevant medications^f^	X	X^f^	X^f^			
Physical examination^h^	X					X
Clinical laboratory assessments^i^	X^j^					X^k^
Physical function and outcomes^m^				X	X	X^oo^
Cognitive function and outcomes^n^				X	X	X^oo^
Organ function (SOFA)^u^		X				
Vital signs^w^	X					X
RASS		X^z^				
Major ICU interventions^bb^			X			
Adverse events^cc^	X	X	X^dd^			X
CAM-ICU-7^ee^		X^z^				
Time of extubation^gg^	X					
Duration of mechanical ventilation			X			X^hh^
Level of care			X			X
Mortality			X	X	X	
ICU Memory Tool				X^ii^		X^oo^
Psychological outcomes^jj^				X	X	
Late onset of drug‑induced liver injury			X			
Quality of life^kk^				X	X	

*ABG* arterial blood gas, *AE* adverse event, *BPI* Brief Pain Inventory, *CAM-ICU-7* 7-point scale of the Confusion Assessment Method for the Intensive Care Unit, *CPOT* Critical Care Pain Observation Tool, *D* day, *ECLS* extracorporeal life support, *Ecrf* electronic case report form, *EOT* end of treatment, *EtCO*_2_ end-tidal carbon dioxide, *EW* early withdrawal, *FAQ* functional activities questionnaire, *FiO*_2_ fraction of inspired oxygen, *h* hour, *ICU* intensive care unit, *IES‑R* impact of event scale, *IQCODE* Informant Questionnaire on Cognitive Decline in the Elderly, *Katz ADL* Katz Index of Independence in Activity of Daily Living, *LAR* legally authorized representative, *LTO* Long-Term Outcomes, *M* month, *P0.1* airway occlusion pressure, *PC* pressure assist/control, *PEEP* positive end‑expiratory pressure, *PIP* peak inspiratory pressure, *PROMIS* Patient-Reported Outcomes Measurement Information System, *PS* pressure support, *Q* every, *RASS* Richmond Agitation Sedation Scale, *SAPS* Simplified Acute Physiology Score, *Sedaconda ACD‑S* Sedaconda Anesthetic Conserving Device – S, *SOC* standard of care, *SOFA* sequential organ failure assessment, *SpO*_2_ peripheral capillary oxygen saturation, *TICS* Telephone Interview for Cognitive Status, *UNS* unscheduled, *W* week(s), *WAIS* Wechsler adult intelligence scale, *WHODAS 2.0* World Health Organization Disability Assessment Schedule 2.0, *WMS* Wechsler memory scale

^a^Major ICU interventions, information on ICU care, and relevant concomitant medications are to be collected at Study Day 30 or at ICU discharge, whichever comes first. Level of care (can be collected retrospectively for the whole study period), duration of mechanical ventilation, late onset drug-induced liver injury, mortality, and follow-up of any unresolved AEs are to be performed through telephone call if not possible to retrieve information through medical records at Study Day 30 (regardless of ICU discharge status)

^b^Patients will be followed-up via telephone call at 3 and 6 months (±4 weeks)

^c^Unless consent is withdrawn, the patient should continue in the study for assessment and follow-up even if study drug is discontinued. If consent is withdrawn, patients will be encouraged to complete the EW visit, if possible

^d^Informed consent must be obtained from patient or patient’s LAR before any study-related procedures are performed. If the patient is unable to consent at the time of Screening, informed consent may be obtained from the patient’s LAR; however, information about the study will be given to, and consent obtained from, the patient as soon as the patient’s condition allows

^e^To be assessed only if Initial Screening is performed more than 24 hours prior to the Complete Screening

^f^All relevant prior and concomitant medications are recorded from ICU admission or 24 hours prior to initiation of study drug treatment, whichever is shortest, until the end of the 24-hour post‑treatment monitoring period. After this, only receipt of specific sedative, antipsychotic, and analgesic medications and relevant concomitant medications associated with ongoing AEs will be collected from 24 hours after EOT until Study Day 30 or ICU discharge, whichever comes first. All administered opioids, including the mean opioid dose assessment at 60 minutes prior to randomization, are to be collected also during Screening

^g^Body weight (lb) and height (inch) should be measured when possible or be estimated. Weight and height available in patient’s records can be used if measured within the last 7 days

^h^Physical examinations should be performed appropriately, per the patient’s condition. Any findings should be recorded on the physical examination eCRF

^i^Includes clinical chemistry, lipid profile, hematology, and coagulation. Screening/Baseline clinical laboratory tests, other than those pertaining to trial eligibility, may be collected at any time during the Complete Screening (-24 hours to 0 hour) or post-randomization period (0 hour to +6 hours) prior to initiation of study drug treatment

^j^Assessment should be performed once during post-treatment monitoring period, if the patient is still in the ICU. Analyses performed per SOC with an 18-to-48-hour window after EOT can be used

^k^Only applicable if EW is during study drug treatment period

^l^Female patients of childbearing potential must have a negative (serum or urine) pregnancy test prior to randomization. A pregnancy test obtained previously as part of usual care during this hospital episode that is documented in the patient’s medical record will suffice. Female patients not of childbearing potential are defined as female patients who have been postmenopausal for at least 1 year, have been surgically sterilized, or are 60 years of age

^m^Physical outcomes assess activities of daily living by the Katz ADL and Pfeffer FAQ

^n^Cognitive baseline will be assessed by the IQCODE. LTO will be assessed by TICS, WAIS IV-Digit Span, Hayling Sentence Completion Test, Controlled Oral Word Association, WMS-IV – Immediate Memory (Adult/Older Adult), WMS-IV – Delayed Memory (Adult/Older Adult), and PROMIS Cognitive Function questionnaire

^o^To be performed within 30 minutes from randomization

^p^SAPS III, reasons for ICU admission, ICU diagnosis criteria, hospital and ICU admission, time for intubation, and exposure of volatile anesthetics in the past 24 hours (and if yes, what drug [sevoflurane/isoflurane/desflurane]) to be assessed at Complete Screening

^q^Predicted remaining time on the ventilator for each patient to be collected just shortly before randomization in order to monitor the proportion of patients with longer (>24 hours) versus shorter (12 to 24 hours) exposure times

^r^Ventilator parameters include ventilator mode, set tidal volume, observed tidal volume, set rate, observed rate, observed minute volume, set PEEP, PS above PEEP, PC above PEEP, PIP, plateau pressure (once daily only), mean airway pressure, FiO_2_, SpO_2_, EtCO_2_, ventilator trigger, P0.1, and ABG

^s^These assessments will be performed more frequently when clinically indicated (as patients will often be observed continuously or more frequently than every 8 hours in clinical practice)

^t^Only applicable when arterial line is available

^u^Organ function will be assessed by SOFA once daily at Baseline, during the study drug treatment period, the 24-hour post-treatment period, and until 7 days after EOT

^v^After randomization, the study equipment will be set-up and study drug treatment shall be initiated as close to randomization as possible and no later than 6 hours after randomization

^w^Vital signs include systolic, diastolic, and mean arterial blood pressure, heart rate, SpO_2_, (measured by pulse oximetry; will not be assessed while patients are on ventilator support, as it will be captured as a ventilator parameter), respiratory rate (will not be recorded as part of vital sign assessments while patient is on ventilator support, as it will be captured in the ventilator parameter records as observed breathing rate), and body temperature

^x^Unblinded baseline assessment for RASS and CPOT should be performed within 30 minutes prior to initiation of study drug administration. Vital signs should be performed within 60 minutes prior to initiation of study drug administration

^y^Assessment will be performed in a blinded manner by a blinded assessor

^z^CAM-ICU-7 and RASS will be performed daily (at a minimum) during the study drug treatment period and until 7 days after EOT or until hospital discharge, whichever comes first. However, more frequent assessments will be performed when clinically indicated. RASS

^aa^A separate gas monitor will be readily available during the study drug treatment period for measurement of end-tidal isoflurane concentrations. Only applicable for isoflurane‑treated patients

^bb^Major ICU interventions through Study Day 30 or until ICU discharge, whichever comes first, include the following: renal replacement therapy, ECLS, tracheostomy, non‑invasive ventilation, and re-admission to the ICU

^cc^Recording of AEs will start at the initiation of study drug administration and continue until Day 7 post EOT

^dd^Only AEs unresolved at D7 to be followed-u

^ee^CAM-ICU-7 will be assessed 60 (±10) minutes after EOT in all patients by a blinded assessor. CAM-ICU-7 will not be required for patients reaching EOT due to treatment failure, patients transitioned to comfort care, or patients continued onto benzodiazepines or propofol sedation due to clinical need before 60 minutes after EOT

^ff^Wake-up test will be assessed through blinded RASS assessments

^gg^To be collected for patients who are extubated on study drug only

^hh^Only applicable if EW is after EOT

^ii^Memory panorama from time in the ICU will be assessed by the ICU Memory Tool at the 3-month follow-up visit only

^jj^Psychological outcomes will be assessed by the PROMIS Depression and Anxiety questionnaires and IES-R

^kk^Quality of life will be assessed by the WHODAS 2.0 and BPI questionnaires

^ll^Baseline data can be collected until 48 hours after initiation of study drug treatment as the time period of interest is not synonymous with when data needs to be captured

^mm^As soon as the study drug treatment has been initiated, the SOC sedative should be stopped (ie, slow weaning will not be permitted) to limit the impact of residual SOC sedation

^nn^For isoflurane-treated patients only

^oo^LTO assessments will be completed at 3 and 6 months. ICU Memory Tool will be assessed at 3 months follow-up only

$$\% of time sedation depth within target=100\%\times \frac{success time}{success time+failure time}$$

**Table 4:** Handling of intercurrent events for the primary estimand

Intercurrent event*	Summary of pre-defined plan for handling
Rescue medication	Following second-line rescue sedation bolus, the 30 min immediately thereafter will be coded as failure time. For second-line rescue sedation continuous infusion, the time from rescue infusion start until 60 min after its discontinuation will be coded as failure time
Intentional lightening of sedation for clinical purposes (e.g., awakening test, neurological assessment)	RASS will be censored for the duration of intentional sedation lightening and for 30 min immediately thereafter
Additional sedation for minor ICU procedures	RASS will be censored for the duration of additional sedation and the 60 min immediately thereafter
Additional sedation or anesthesia for procedures outside the ICU	Study drug is replaced by standard of care sedation if the participant leaves the ICU during the treatment period. Time from leaving the ICU until the first blinded RASS after return and restart of study treatment will be censored
Neuromuscular blocking agent (NMBA)	For NMBA bolus, the 2 h immediately thereafter will be censored. For NMBA continuous infusion, the time during the infusion and 4 h immediately thereafter will be censored
Treatment discontinuation or death	If related to study drug, treatment discontinuation or death will be coded as failure for the remainder of time from end of treatment through hour 48. For all other treatment discontinuations or death, the calculation of time of adequate sedation depth will stop at end of treatment without penalty or censoring
RASS assessment outside scheduled visit window	Blinded RASS is to be assessed every 2 ± 0.5 h, such that consecutive compliant assessments could be as much as 3 h apart. For RASS assessments separated by > 3 h, the time exceeding 3 h will be used for coding the mid-part of the interval as censored
Missing blinded RASS assessment	RASS will be censored from the scheduled timepoint to the midpoints between the scheduled timepoint and adjacent RASS assessments. For example, if blinded RASS is performed in precisely 120-min intervals, the censored period will be 60 min before and 60 min after the missed assessment

^*^ Intercurrent events are those that have potential to preclude observation of the outcome or affect its interpretation. Additional details regarding computation of success, failure, and censoring times, with illustrative hypothetical examples, are provided in the statistical analysis plan

Percentage of time at the target sedation depth was selected as the primary outcome given the trial objective of evaluating isoflurane for the novel indication of sedation for ICU patients. RASS is routinely used in clinical practice to assess sedation depth and to prescribe a target level of sedation to which the sedative drug is titrated. Similar to prior sedation trials [4, 7], the RASS target includes 4 steps. Permissible RASS ranges from RASS − 1, characterized as drowsy, not fully alert but has sustained awakening > 10 s to voice, to RASS − 4, characterized as deep sedation with no response to voice but movement or eye opening to physical stimulation. The allowable RASS range excludes coma given the established efficacy of isoflurane for producing coma as an approved agent for general anesthesia.

Utilizing the estimand framework established by the International Council for Harmonisation of Technical Requirements for Pharmaceuticals for Human Use (ICH E9 R1), the primary estimand is further characterized by the following:

- Population: Adult ICU patients anticipated to have clinical need for > 12 h of invasive ventilation and continuous sedation for RASS − 1 to − 4, as per trial eligibility criteria.
- Primary endpoint: The percentage of time sedation depth is maintained within RASS − 1 to − 4 during study treatment.
- Intercurrent events: Rescue medication, intentional lightening of sedation for clinical purposes, additional sedation for procedures, neuromuscular blockade, treatment discontinuation, death, RASS assessment outside scheduled visit window, and missing blinded RASS assessment. Handling of intercurrent events is summarized in Table 4.
- Population-level summary: The absolute difference between treatment groups in mean percentage of time sedation is maintained within the target range.

### Secondary outcomes

The trial has four key secondary outcomes. Effect on opioid use will be evaluated as the change in mean fentanyl-equivalent opioid dose (Additional File 1) during the study treatment period compared to the mean opioid dose in the 60 min prior to protocol-directed opioid reduction. Wake-up time after end of treatment will be evaluated as the time from study drug cessation to achieving a blinded RASS ≥ 0 in participants for whom spontaneous awakening is not contraindicated. Cognitive recovery will be evaluated as the CAM-ICU-7 score, a 7-point scale, assessed 60 (± 10) min after end of treatment in patients not re-sedated with benzodiazepine or propofol infusions [17]. Effect on spontaneous breathing effort will be evaluated as the proportion of ventilator parameter assessments in which spontaneous breathing effort is present during the treatment period. Spontaneous breathing effort will be deemed present if any of the following are met: airway occlusion pressure (P0.1) > 0 cm H_2_O, use of pressure support or another spontaneous ventilator mode, or observed respiratory rate exceeds set respiratory rate. Additional secondary outcomes are detailed in Table 5.

**Table 5:** INSPiRE-ICU2 secondary and exploratory outcomes

Objectives	Endpoints
**Key secondary outcomes**	
To compare the effect of isoflurane vs propofol on use of opioids during the study drug treatment period	Change in mean fentanyl-equivalent opioid dose during the study drug treatment period compared to mean opioid dose 60 min prior to protocol-directed opioid reduction
To compare the effect of isoflurane vs propofol on the wake up time at end of study drug treatment	Time from stop of study drug treatment to blinded RASS ≥ 0, up to 4 h
To compare the effect of isoflurane vs propofol on cognitive recovery after end of treatment	CAM-ICU-7 score assessed 60 (± 10) min after end of treatment in patients not re-sedated with benzodiazepine or propofol infusions
To compare the effect of isoflurane vs propofol on spontaneous breathing effort during the study drug treatment period	Proportion of ventilator parameter observations with spontaneous breathing efforts during the study drug treatment period
**Other secondary outcomes**	
To compare the effect of isoflurane vs propofol on time from sedation termination to extubation in patients for whom study drug is terminated for extubation	Time from end of treatment to extubation if study drug is terminated for extubation
To compare the effect of isoflurane vs propofol on days alive and free of mechanical ventilation through study day 30	Days alive and free of invasive mechanical ventilation through study day 30
To compare the effect of isoflurane vs propofol on days alive and free of the ICU	Days alive and free of the ICU through study day 30
To compare the effect of isoflurane vs propofol on delirium and coma free days until 7 days after end of treatment	Delirium and coma free days from start of study drug until 7 days after the end of treatment, as assessed with CAM-ICU and RASS
To compare the effect of isoflurane vs propofol on mortality at 30 days after randomization	Mortality rate at 30 days after randomization
To compare the effect of isoflurane vs propofol on mortality at 3 months after randomization	Mortality rate at 3 months after randomization
To compare the effect of isoflurane vs propofol on mortality at 6 months after randomization	Mortality rate at 6 months after randomization
To compare the safety of isoflurane vs propofol	Adverse events, clinical laboratory assessments, vital signs, physical examination, blood gases, organ function, urinary output, and ventilator parameters
To assess ACD-S device deficiencies in patients receiving isoflurane	Frequency and type of ACD-S device deficiencies in patients receiving isoflurane
To compare the use of restraints in patients receiving isoflurane vs propofol	Proportion of patients using restraint during the study drug treatment period
**Exploratory**	
To assess isoflurane dose over time	• Isoflurane dose in mL/hour; • Isoflurane dose in mL/hour/L minute ventilation; and • End-tidal isoflurane concentration every 4 h
To compare the effect of isoflurane vs propofol on major ICU interventions through study day 30 or until ICU discharge, whichever comes first	Need for: • Renal replacement therapy; • ECLS; • Tracheostomy; and • Non-invasive ventilation
To compare the effect of isoflurane vs propofol on level of care up to 30 days after randomization	Level of care: • Patient deceased; • Still in ICU; • Intermediary care unit; • General ward; • Another ICU (within or outside the hospital); • Another hospital (unknown ward); • Rehabilitation unit; • Nursing home; and • Home
To assess the end tidal isoflurane concentration and relation to RASS scores	End-tidal isoflurane concentration over time and relation to RASS scores
To compare oxygenation (PaO_2_/FiO_2_) over time during the study drug treatment period, in patients with acute respiratory distress syndrome and acute hypoxemic respiratory failure, in isoflurane-vs-propofol-treated patients	Oxygenation (PaO_2_/FiO_2_) in patients with acute respiratory distress syndrome and acute hypoxemic respiratory failure over time during the study drug treatment period
To compare memory panorama from time in ICU in isoflurane-vs-propofol treated patients	Number of factual memories, memories of feelings, or delusional memories, as assessed by the ICU Memory Tool, collected at 3 months post-randomization
To compare physical outcomes at 3 and 6 months post-randomization in isoflurane-vs-propofol-treated patients	Activities of daily living, as assessed by the Katz Index of Independence in Activity of Daily Living and Pfeffer Functional Activities Questionnaire, at 3 and 6 months post-randomization
To compare psychological outcomes at 3 and 6 months post-randomization in isoflurane-vs-propofol-treated patients	Depression, anxiety, and post-traumatic stress symptoms, as assessed by Impact of Event Scale (IES-R) and PROMIS Depression and Anxiety questionnaires, at 3 and 6 months post-randomization
To compare cognitive function 3 and 6 months post-randomization in isoflurane-vs-propofol-treated patients	Cognitive function, as assessed by Telephone Interview for Cognitive Status (TICS), Wechsler adult intelligence scale IV-Digit Span/Immediate Memory/Delayed Memory, Hayling Sentence Completion Test, Controlled Oral Word Association, and Patient-Reported Outcomes Measurement Information System 2.0, at 3 months and 6 months post-randomization
To compare quality of life at 3 and 6 months post-randomization in isoflurane-vs-propofol-treated patients	Quality of life at 3 and 6 months post-randomization, as assessed by World Health Organization Disability Assessment Schedule 2.0 and Brief Pain Inventory
To compare the need for rescue, other sedatives, and antipsychotics in isoflurane-vs-propofol-treated patients	Use of rescue sedatives, other sedatives, and antipsychotics from randomization to end of treatment
To compare the hemodynamic instability in isoflurane-vs-propofol-treated patients	Change from baseline in vasopressor requirement (norepinephrine equivalent mean dose)
To compare duration of mechanical ventilation in isoflurane-vs-propofol treated patients	Duration of mechanical ventilation
To compare ICU length of stay in isoflurane-vs-propofol-treated patients	ICU length of stay
To compare minute ventilation in isoflurane-vs-propofol treated patients	Change in minute ventilation during study drug treatment period every 4 h during study drug treatment

### Protocol compliance

To ensure protocol compliance, all dose titrations of study medication and the indication for change will be recorded. Study teams will maintain communication with clinical staff to promote adherence to protocol, including verification that blinding materials are in place before blinded assessments are conducted. Blinded assessors will attest to having received training on performance of RASS and CPOT, and to lack of knowledge of study treatment allocation with each blinded RASS assessment. If a blinded assessor becomes aware of treatment group, they will not conduct any further blinded assessments for that participant.

### Safety monitoring

Surveillance for and reporting of adverse events (AEs) will occur through the 7-day post-treatment follow-up period, and any AEs occurring during this period will be followed until returned to normal, stabilized, or no longer clinically significant. Adverse events of special interest (AESIs) are specified in Table 6.

**Table 6:** Adverse events of special interest (AESIs)

**AESI**	**Mild**	**Moderate**	**Severe**	**Serious AE (SAE)**
Hypoxemia	Oxygen desaturation event that requires an increase in FiO_2_ of >10% or any increase in PEEP for >60 min to maintain SpO_2_ of at least 88%, despite ventilator optimization.	Oxygen desaturation event that requires an increase in FiO_2_ >20% or increase in PEEP of >5 cmH_2_O for >60 min to maintain SpO_2_ of at least 88%, despite ventilator optimization.	Refractory hypoxemia, defined as SpO_2_ <88% lasting for 30 min or longer, despite ventilator optimization.	Need for respiratory rescue therapy, defined as ECMO, ECCO2R, inhaled nitric oxide, or inhaled epoprostenol initiated for life threatening refractory hypoxemia, or other life-threatening manifestations of hypoxemia.
Hypercapnia	PCO2 10 to 15 mmHg above baseline on 2 consecutive blood gases at least 60 min apart, despite ventilator optimization.	pCO2 16 to 20 mmHg above baseline on 2 consecutive blood gases at least 60 min apart, despite ventilator optimization.	pCO2 >20 mmHg above baseline on 2 consecutive blood gases at least 60 min apart, despite ventilator optimization.	ECMO support or life-threatening manifestations of respiratory acidosis.
Malignant hyperthermia (MH)				Any episode of MH, which may be characterized by muscle rigidity; unexplained hypercapnia resistant to increasing minute ventilation; elevated CK or urine myoglobin suggesting rhabdomyolysis; acute hyperkalemia >6 mmol/L potentially resulting in ECG changes of peaked T-waves, increased ventricular tachycardia, or ventricular fibrillation and hyperthermia in a patient exposed to volatile anesthetic or succinylcholine.
Propofol infusion syndrome (PRIS) suspected by Investigator				Any episode of PRIS, characterized by the development of otherwise unexplained metabolic acidosis and cardiac dysfunction with at least 1 of rhabdomyolysis, hypertriglyceridemia, or renal failure after initiation of propofol.
Accidental self-extubation	Accidental self-extubation will be recorded as an AESI, but grading is unnecessary, per FDA guidance			
Hypotension	New episode of SBP <90* mmHg or MAP <65* mmHg lasting at >60 min **OR **fluid bolus ³1000 mL over <60 min **OR **new low dose vasopressor <0.05 mcg/kg/min norepinephrine equivalent >60 min **OR **increase of existing vasopressor by 0.05 to 0.1 mcg/kg/min norepinephrine equivalent over from baseline and increase lasting >60 min.	New low-dose vasopressor 0.05 to <0.2 mcg/kg/min norepinephrine equivalent OR increase over <60 min of vasopressor(s) by 0.1 to <0.2 mcg/kg/min norepinephrine equivalent and lasting >60 min, to maintain SBP ³90* mmHg or MAP ³65* mmHg.	New vasopressor ³0.2 mcg/kg/min norepinephrine equivalent or increase over <60 min of vasopressor(s) by ³0.2 mcg/kg/min norepinephrine equivalent and lasting >60 min, to maintain SBP ³90* mmHg or MAP ³65* mmHg.	Immediate life-threatening hypotension, requiring intervention, e.g., CPR, mechanical circulatory support.
Liver injury	1) ALT ³3 ´ ULN; or AST ³3 ´ ULN; or total bilirubin >2 ´ ULN; or alkaline phosphatase >2 ´ ULN.	Hy’s Law criteria (ALT or AST 3 ´ ULN and total bilirubin >2 ´ ULN).	Mild encephalopathy and Hy’s Law criteria (ALT or AST 3 ´ ULN and total bilirubin >2 ´ ULN).	Life-threatening consequences; moderate to severe encephalopathy; coma and Hy’s Law criteria (ALT or AST >3 ´ ULN and total bilirubin >2 ´ ULN).
Hyperkalemia	>5.5 mEq/L (>5.5 mmol/L) in a non-hemolyzed sample and in absence of respiratory acidosis.	>6.0 to 6.5 mEq/L (6.0 to 6.5 mmol/L) in a non-hemolyzed sample for which a medication or dialysis to lower potassium was prescribed.	>6.5 to 7.0 mEq/L (>6.5 to 7.0 mmol/L) in a non-hemolyzed sample for which a medication or dialysis to lower potassium was given.	>7.0 mEq/L (>7.0 mmol/L) in a non-hemolyzed sample for which a medication or dialysis to lower potassium was prescribed, or life-threatening arrhythmia due to hyperkalemia.
Rhabdomyolysis independent of MH	CK 10,000 to 20,000 U/L	CK >20.000 U/L with moderate renal failure graded as AKIN Stage 3.	CK >20.000 U/L requiring dialysis.	Life-threatening consequences of rhabdomyolysis.

*Unless a different clinical target is selected by the clinical team prior to randomization

*AESI* adverse event of special interest, *AKIN* Acute Kidney Injury Network, *ALT* alanine aminotransferase, *AST* aspartate aminotransferase, *CK* creatine kinase, *CPR* cardiopulmonary resuscitation, *ECCO2R* extracorporeal carbon dioxide removal, *ECG* electrocardiogram, *ECMO* extracorporeal membrane oxygenation, *FDA* Food and Drug Administration, *FiO*_2_ fraction of inspired oxygen, *MAP* mean arterial pressure, *MH* malignant hyperthermia, *pCO*_2_ partial pressure of carbon dioxide, *PEEP* positive end-expiratory pressure, *PRIS* propofol-related infusion syndrome, *SAE* serious adverse event, *SBP* systolic blood pressure, *SpO*_2_ peripheral capillary oxygen saturation, *ULN* upper limit of normal

An independent Data Safety Monitoring Board (DSMB) will review data for both this trial (NCT05327296) and the companion trial (NCT05312385) being conducted under the same US Food and Drug Administration (FDA) Investigational New Drug application. The DSMB review will focus on safety; efficacy data will not be reviewed. Scheduled meetings will occur at 25%, 50%, and 75% information fraction for the 30-day follow-up period. An ad hoc DSMB meeting also may be called if interval safety concerns arise.

### Data management

Protection of confidentiality will be maintained at every step of the enrollment and participation process in accordance with Good Clinical Practice (GCP) principles and the US Health Insurance Portability and Accountability Act (HIPAA). Enrolled participants will each be assigned a unique identifier under which any data related to the participant will be shared with the sponsor and entered into a secure web-based electronic data capture (EDC) platform. Documentation that contains patient identifiers, such as signed informed consent forms, will be maintained by site investigators in a secure location for the duration of the trial and for the time specified by clinical trial agreements after the conclusion of the trial. Quality control will include EDC logic checks and direct source verification from independent trial monitors. Changes to entered data will be tracked by an audit trail. The primary investigator at each site will attest to accuracy and completeness of the final dataset at their site.

### Sample size

A non-inferiority margin of 15% is set for efficacy of isoflurane versus propofol to maintain target sedation depth. This margin was chosen based on the calculation that failing to reach the sedation target on 2 assessments (spanning approximately 4 h) in a 24-h period with isoflurane compared to propofol would translate to a 15% absolute difference, which we deemed clinically relevant. Enrollment of 235 randomized participants yields 95% power for the non-inferiority test with 1-sided α 0.025 assuming 5% attrition.

### Data analysis

The primary efficacy analysis will be conducted on the intent-to-treat (ITT) analysis set, which includes all randomized participants who receive any amount of study medication. A modified intent-to-treat (mITT) analysis set will also be employed for all efficacy analyses and include patients who have had ≥ 6 h of study sedation and at least 3 blinded RASS assessments. Efficacy analysis will also be conducted on a per-protocol (PP) analysis set that includes randomized participants who have received ≥ 8 h of study sedation with ≥ 50% of planned RASS assessments performed and no major protocol deviation affecting the primary efficacy analysis. The safety analysis set will include all participants in both the run-in and randomization phases who receive any amount of the study medication.

The primary efficacy endpoint, the percentage of time at target RASS, without rescue sedation will be analyzed in a non-inferiority test utilizing the analysis of variance (ANOVA) model that includes treatment group, SAPS-III, and surgical status as fixed effects. Handling of missing and mistimed RASS assessments is detailed above in the primary estimand formulation. Key secondary efficacy endpoints will be tested via a superiority test in a hierarchical manner. In this way, key secondary endpoints will require statistical significance of the previous secondary endpoint and the primary efficacy endpoint in favor of the participants who received isoflurane. No interim efficacy analysis will be conducted. Long-term follow-up data will be analyzed separately once all patients complete the 6-month follow-up visit.

### Reproducibility of trial results

Trial results will be published in a peer-reviewed academic medical journal. A second, identically designed trial known as INSPiRE-ICU1 (NCT05312385) is also being conducted in the US. INSPiRE-ICU1 and INSPiRE-ICU2 have no overlap in enrolled patients or recruiting sites. Execution of two identically designed trials will help determine reproducibility of trial results and bolster interpretation of data. Final results of this trial will be submitted to regulatory authorities within 12 months after completion of the study. Trial information and results will also be made publicly available on clinicaltrials.gov. Ownership of the dataset and statistical code will remain proprietary to the trial sponsor, Sedana Medical.

## Discussion

The overall goal of INSPiRE-ICU2 is to evaluate the efficacy of inhaled isoflurane, compared to intravenous propofol, for sedation in mechanically ventilated ICU patients. Along with the identically designed INSPIRE ICU 1 trial, which was conducted simultaneously, this trial is the first to systematically evaluate volatile anesthetics for ICU sedation in the US and entails the use of a novel device tying respiration to sedation and, for many critical care clinicians, a new sedative agent in isoflurane. It is designed to rigorously address several pressing questions about isoflurane: efficacy as a sedative, safety, potential opioid-sparing effect, and impact on wake-up time, cognitive recovery, and spontaneous breathing.

The first critical question for this trial is efficacy of isoflurane as a sedative. The allowable sedation range excludes patients requiring an unarousable state (RASS −5), since efficacy of isoflurane for general anesthesia is well established, as well as any fully awake/alert state (RASS ≥ 0). A recent multicenter European randomized trial found isoflurane and propofol achieved near-identical proportions of time within sedation target, but RASS assessments were unblinded [7]. To mitigate bias, INSPiRE-ICU2 requires RASS assessments for the primary endpoint be performed by trained individuals blinded to treatment assignment and uninvolved in the patient’s care. Additionally, we adopt the estimand framework to account for intercurrent events that could preclude observation of the primary outcome or affect its interpretation, lending additional rigor.

Second, INSPiRE-ICU2 evaluates the effect of isoflurane versus propofol on concomitant opioid exposure. Isoflurane may have direct analgesic effects that diminish need for opioid analgesics. In the aforementioned European trial, isoflurane was associated with lower opioid requirements compared to propofol [7]. To mitigate potential bias in clinicians hypothesizing an opioid-sparing isoflurane effect, INSPiRE-ICU2 protocolizes halving continuous-infusion opioids in both treatment arms prior to initiation of study sedative, with subsequent titration guided by a clinically validated pain scale. Change in fentanyl-equivalent opioid dose during the treatment period, compared to the 60 min prior to randomization, will be used to quantify potential opioid-sparing effect and paired with independent blinded assessments of pain to ensure unbiased evaluation in both groups.

Third, the impact of isoflurane versus propofol on cognitive recovery 1 h after end-of-treatment will be assessed. Limited data suggest volatile anesthetics for ICU sedation may be associated with less delirium upon discontinuation than other sedatives [18], but this has not been tested rigorously. Cognitive recovery will be assessed in patients not requiring re-sedation via the CAM-ICU-7, again conducted in blinded fashion to mitigate risk of bias. Additional neurocognitive testing will be conducted in a subset of survivors at 3 and 6 months.

Finally, INSPiRE-ICU2 will evaluate effects of isoflurane versus propofol on spontaneous breathing effort. Propofol, benzodiazepines, and opioids have a respiratory depressant effect termed respirolysis [19], though a subset of patients appear to have refractory high respiratory drive despite deep sedation [20]. Existing data suggest isoflurane better preserves spontaneous breathing [21], which is potentially relevant to avoid diaphragm disuse atrophy as part of a lung- and diaphragm-protective ventilation strategy [22] that may accelerate respiratory recovery.

Important questions will remain unanswered by this trial. Study isoflurane exposure will not exceed 54 h per protocol, leaving unaddressed potential beneficial or untoward effects of longer exposure. This treatment length was chosen to evaluate safety and efficacy with moderate duration of use. Though not tested in the parent trial, off-target molecular effects of isoflurane and other volatile anesthetics might confer lung protection in at-risk patients [23, 24]. In preclinical models, isoflurane decreases inflammatory cell recruitment into the lungs, attenuates release of inflammatory mediators following proinflammatory insults [25, 26], decreases lung barrier permeability, and improves lung function following injury with endotoxin [27] or during injurious mechanical ventilation [28]. Few studies have investigated the immunomodulatory effects of isoflurane or other volatile anesthetics in critically ill ICU patients. A small pilot trial in ARDS patients demonstrated sevoflurane decreased bronchoalveolar lavage and plasma levels of alveolar epithelial injury and improved oxygenation [29]. Further studies are needed to determine whether isoflurane and other volatile anesthetics indeed prevent or facilitate recovery from acute lung injury. A prospective biospecimen substudy conducted at a subset of sites within INSPiRE-ICU2 and INSPiRE-ICU1 will help address this question for isoflurane. Additionally, our trial will not provide any information as to the environmental impact of isoflurane sedation as compared to propofol.

In conclusion, INSPiRE-ICU2 is a phase 3, multicenter trial being conducted in the US to evaluate efficacy and safety of isoflurane, compared to propofol, for continuous sedation of ICU patients requiring invasive mechanical ventilation. Unique design features, including blinded assessments of sedation depth, pain, and cognitive recovery, and protocolized reduction of sedatives/opioids prior to study treatment, ensure a rigorous evaluation of isoflurane in a novel clinical environment—US intensive care units. Trial results have potential to transform the ICU sedation landscape by adding a well-known agent with several appealing properties that circumvent many limitations of other commonly prescribed sedatives in a new setting.

## Trial status

Enrollment began June 30, 2022, and is expected to be completed by May 31, 2024. As of the writing of this manuscript, INSPiRE-ICU2 is being conducted under protocol version 6.0, made available October 6, 2023. Sedana Medical (the study sponsor) or their designated clinical research organization (CRO) will communicate IRB-approved protocol revisions, amendments, and other modifications to all participating sites’ primary investigators and study personnel via email. Clear instructions will be provided at the time of these communications regarding any requirements for the notification of local site IRBs. Details on approved amendments requiring re-consenting of participants or legally authorized representative will be explicitly communicated.

Any revisions will be detailed in the primary results manuscript.

## Supplementary Information

Additional file 1.

Additional file 2.

Additional file 3.

## Data Availability

Sedana Medical (sponsor) is the sole owner of results from the trial. No data can be shared or published before written approval from the sponsor.
