# Supplementary material for: Inhaled sedation versus propofol in respiratory failure in the ICU (INSPiRE-ICU2): study protocol for a multicenter randomized controlled trial
Source: Trials. 2025 Mar 31;26:114. doi: 10.1186/s13063-025-08791-0 (PMC11956472; doi:10.1186/s13063-025-08791-0)
Supplement: Supplementary file 1 — Additional file 1. [file 13063_2025_8791_MOESM1_ESM.pdf]

ADDITIONAL FILE 1: Opioid Dose Calculation to Determine IV Fentanyl Equivalents

The intravenous fentanyl-equivalent opioid dose will be used to compare opioid doses when different agents are chosen by the clinical team. The fentanyl-equivalent opioid dose will be calculated via the following 3 steps:

1. Oral opioids will be converted to oral morphine equivalents according to Table E1.
2. Oral morphine equivalents will be converted to intravenous equivalents (formula below)
3. Intravenous fentanyl-equivalents will be calculated per the intravenous opioid conversion factors in Table E2.

Table E1: Opioid Oral Morphine Milligram Equivalent (MME) Conversion Factors[1,2]

**Type of Opioid (strength units)**	**MME Conversion Factor**
Buprenorphine film/tablet[3] (mg)	30
Buprenorphine patch[4] (mcg/hr)	12.6
Buprenorphine film (mcg)	0.03
Butorphanol (mg)	7
Codeine (mg)	0.15
Dihydrocodeine (mg)	0.25
Fentanyl buccal or SL tablets, or lozenge/troche[5] (mcg)	0.13
Fentanyl film or oral spray[6] (mcg)	0.18
Fentanyl nasal spray[7] (mcg)	0.16
Fentanyl patch[8] (mcg)	7.2
Hydrocodone (mg)	1
Hydromorphone (mg)	4
Levorphanol tartrate (mg)	11
Meperidine hydrochloride (mg)	0.1
Methadone[9] (mg)	
>0, ≤20	4
>20, ≤40	8
>40, ≤60	10
>60	12
Morphine (mg)	1
Opium (mg)	1
Oxycodone (mg)	1.5
Oxymorphone (mg)	3
Pentazocine (mg)	0.37
Tapentadol[10] (mg)	0.4
Tramadol (mg)	0.1

1. The MME conversion factor is intended only for analytic purposes where prescription data is used to calculate daily MME. It is to be used in the formula: Strength per Unit × (Number of Units/Days Supply) × MME conversion factor = MME/Day. This value does not constitute clinical guidance or recommendations for converting patients from one form of opioid analgesic to another. Please consult the manufacturer's full prescribing information for such guidance. Use of this file for the purposes of any clinical decision-making warrants caution.
2. National Center for Injury Prevention and Control. CDC compilation of benzodiazepines, muscle relaxants, stimulants, zolpidem, and opioid analgesics with oral morphine milligram equivalent conversion factors, 2016 version. Atlanta, GA:

Centers for Disease Control and Prevention; 2016. Available at https://www.cdc.gov/drugoverdose/media/.

3. Buprenorphine formulations with an FDA approved indication for MAT are excluded from Medicare's Overutilization Monitoring System's opioid overutilization reporting.
4. The MME conversion factor for buprenorphine patches is based on the assumption that one milligram of parenteral buprenorphine is equivalent to 75 milligrams of oral morphine and that one patch delivers the dispensed micrograms per hour over a 24-hour day. Example: 5 μg/hr buprenorphine patch × 24 hours = 120 μg/day buprenorphine = 0.12 mg/day = 9 mg/day oral MME. In other words, the conversion factor not accounting for days of use would be 9/5 or 1.8. However, since the buprenorphine patch remains in place for 7 days, we have multiplied the conversion factor by 7 (1.8 × 7 = 12.6). In this example, MME/day for four 5 μg/hr buprenorphine patches dispensed for use over 28 days would work out as follows: Example: 5 μg/hr buprenorphine patch × (4 patches/28 days) × 12.6 = 9 MME/day. Please note that because this allowance has been made based on the typical dosage of one buprenorphine patch per 7 days, you should first change all Days Supply in your prescription data to follow this standard, ie, Days Supply for buprenorphine patches = # of patches × 7.
5. The MME conversion factor for fentanyl buccal tablets, sublingual tablets, and lozenges/troche is 0.13. This conversion factor should be multiplied by the number of micrograms in a given tablet or lozenge/troche.
6. The MME conversion factor for fentanyl film and oral spray is 0.18. This reflects a 40% greater bioavailability for films compared to lozenges/tablets and 38% greater bioavailability for oral sprays compared to lozenges/tablets.
7. The MME conversion factor for fentanyl nasal spray is 0.16, which reflects a 20% greater bioavailability for sprays compared to lozenges/tablets.
8. The MME conversion factor for fentanyl patches is based on the assumption that one milligram of parenteral fentanyl is equivalent to 100 milligrams of oral morphine and that one patch delivers the dispensed micrograms per hour over a 24 hour day. Example: 25 μg/hr fentanyl patch × 24 hours = 600 μg/day fentanyl = 60 mg/day oral morphine milligram equivalent. In other words, the conversion factor not accounting for days of use would be 60/25 or 2.4. However, since the fentanyl patch remains in place for 3 days, we have multiplied the conversion factor by 3 (2.4 × 3 = 7.2). In this example, MME/day for ten 25 μg/hr fentanyl patches dispensed for use over 30 days would work out as follows:
   Example: 25 μg/hr fentanyl patch × (10 patches/30 days) × 7.2 = 60 MME/day. Please note that because this allowance has been made based on the typical dosage of one fentanyl patch per 3 days, you should first change all Days Supply in your prescription data to follow this standard, ie, Days Supply for fentanyl patches = # of patches × 3.
9. https://www.cdc.gov/drugoverdose/pdf/calculating_total_daily_dose-a.pdf.
10. Tapentadol is a mu receptor agonist and norepinephrine reuptake inhibitor. Oral MMEs are based on degree of mu-receptor agonist activity, but it is unknown if this drug is associated with overdose in the same dose-dependent manner as observed with medications that are solely mu receptor agonists.

CDC = Center for Disease Control; FDA = Food and Drug Administration; MAT = Medication Assisted Treatment; MME = morphine milligram equivalent.

Source: Maryland Department of Health. Opioid Oral Morphine Milligram Equivalent (MME) Conversion Factors. Maryland.gov. https://mmcp.health.maryland.gov/healthchoice/opioid-dur-workgroup/Documents/opioid_morphine_eq_conversion_factors_april2017.pdf. Accessed 25 February 2021.

Conversion of Oral Morphine Equivalents to IV Morphine Equivalents (mcg)

Intravenous morphine equivalents = Oral morphine milligram equivalent × 0.33

Table E2: Intravenous Fentanyl-Equivalent Conversion Factors

Fentanyl Equivalents (mcg)	Conversion Factor
Fentanyl	1
Remifentanil	1
Alfentanil	0.33
Sufentanil	0.1
Morphine	20
Hydromorphone	133
Methadone	25
