# Supplementary material for: Inhaled sedation versus propofol in respiratory failure in the ICU (INSPiRE-ICU2): study protocol for a multicenter randomized controlled trial
Source: Trials. 2025 Mar 31;26:114. doi: 10.1186/s13063-025-08791-0 (PMC11956472; doi:10.1186/s13063-025-08791-0)
Supplement: Supplementary file 2 — Additional file 2. [file 13063_2025_8791_MOESM2_ESM.pdf]

[redacted] University Medical Center ([redacted]UMC)

Consent Form to Participate in a Research Study and HIPAA Authorization

Study title: INSPiRE ICU-2: INhaled Sedation versus Propofol in Respiratory failurE in the ICU
Study number: [redacted] **Approximate number of research participants:** 300 participants ([redacted] at [redacted]
Sponsor: Sedana Medical **Principal Investigator:** [redacted] **Phone:** [redacted]

Note: This consent form is written to address the research participant. If you will be providing consent as a legally authorized representative, the word 'you' should be read as 'the research participant.'

KEY INFORMATION	
Purpose	To determine efficacy and safety of inhaled isoflurane delivered via the Sedaconda ACD-S, compared to intravenous propofol, for sedation in mechanically ventilated ICU adult patients.<br><br>Both medications have been used for anesthesia during surgery for over 25 years in the US and are considered safe and effective. Potential side-effects are well known. Propofol also has been used for sedation during mechanical ventilation in intensive care unit (ICU) patients in the US for decades. Isoflurane has not been used in ICUs in the US due to need for specialized equipment to administer it outside the operating room. This equipment has been approved in Europe (where isoflurane is commonly used in ICUs) but not yet in the US.
Duration	Up to 6 months, including long-term follow-up.
Procedures	You will receive either inhaled isoflurane or intravenous propofol for sedation for up to 54 hours as needed while on the ventilator. You also will have blood drawn for monitoring while in the hospital, typically as part of your routine morning labs. You will receive a phone/video call 3 and 6 months after hospital discharge.
Risks	For both medications, potential side-effects include: decrease in blood pressure, heart rate, or breathing effort. Serious side-effects, such as severe allergic reaction, are very rare. You will be monitored for side effects durjng the study, and the medication dose may be adjusted or stopped as appropriate if you experience any such effects. Additional information on potential risks is provided in the *Section 8: What are the risks of participating in this study?*
Benefits	You may or may not benefit directly from this study. Closer monitoring from study participation could ensure sedation is more precisely adjusted to within the range prescribed by the treating physician. Isoflurane might decrease the need for opioids (pain medication) while on the ventilator. Participants receiving isoflurane might wake up faster once sedation is stopped. Results from this study will benefit future patients by helping identify best options for sedation in ventilator-dependent ICU patients.
Alternatives	Participation is voluntary. You can choose not to take part in this study. If you decide to join the study, you may withdraw from the study at any time. You will continue to receive usual medical care even if you choose not to be in this study or withdraw from this study.
If you are interested in learning more about this study, please read the details below.	

1. Why are you being asked to participate in this research study?

You are being invited to take part in a research study. Before agreeing, it is important that you read and understand why this research is being done and what it will involve for you. This form describes the purpose, procedures, benefits, risks, discomforts, and precautions of the study. It also describes the alternative procedures that are available to you and your right to withdraw from the study at any time.

A member of the study staff will review this form with you and explain the study to you. Please read this form carefully and ask any questions you may have. You can discuss this information with your doctors, family, or anyone else you would like before making your choice.

[redacted]

2. What is the background and purpose of the study?

You are being asked to participate in this research study because you will likely be on a ventilator in the Intensive Care Unit (ICU) for more than 12 hours and require continuous sedation as part of your normal medical care. The ventilator is used to assist your breathing while sedation is used to keep you comfortable on the ventilator. Sedation can range from minimal sedation (drowsy and relaxed) to deep sedation (unconscious and not awakened by verbal stimulation).

Sedation is commonly achieved using medications such as propofol, dexmedetomidine, benzodiazepines, or opioids administered through an intravenous catheter (IV; a tube in your vein) into the bloodstream. These sedatives, given as part of your routine medical care, help to maintain comfort and safety, but they can have side effects, including longer time to wake up, confusion, lower blood pressure and heart rate, and change in breathing pattern.

Another method to provide sedation involves breathing inhaled medications such as isoflurane through the ventilator. Isoflurane is commonly used to provide general anesthesia during surgery, in the United States and around the world. It may allow faster wake-up, less need for pain medications, and earlier return to more normal breathing patterns when compared to IV sedation. Traditionally, isoflurane administration has required large, specialized equipment called an anesthesia machine, limiting its use to operating rooms. A small device, placed in the ventilator breathing circuit, was developed to permit administration of isoflurane without an anesthesia machine, enabling administration in the ICU environment. This adaptor, called the Sedaconda Anesthetic Conserving Device - S (Sedaconda ACD-S) is approved for use in Europe, Australia, Canada, Japan, and other countries, and isoflurane via the Sedaconda ACD-S is routinely prescribed as sedation for ICU patients in these countries. Isoflurane delivered by the Sedaconda ACD-S is approved for ICU sedation in several European countries, including Germany and France, but is not currently approved in the United States. As such, the use of isoflurane for ICU sedation delivered by the Sedaconda ACD-S in this research study is considered investigational.

The purpose of this study is to compare the effects of inhaled isoflurane sedation delivered via the Sedaconda ACD-S with the standard of care IV sedative propofol.

3. Do you have to take part in the study?

Taking part in this research study is voluntary and entirely up to you. You will have to sign and date the consent page within this consent form to indicate you choose to take part. You may change your mind and withdraw without giving any reason, at any time. If you choose to not participate or you withdraw from the study, you will not lose any medical benefits to which you are entitled, and it will not have any effect on your future medical care. You may decide to stop taking part in the study at any time by notifying the study doctor of your decision. If you have some unresolved health problems when you leave the study, the study doctor may, if you agree, need to collect information about your health until the problem resolves.

4. What are your other options should you decide not to take part in the study?

You do not need to take part in this study. If you do not participate, you will receive standard of care sedation as prescribed by your doctors.

5. How many people will be in the study and how long will you be in this study?

Approximately 300 participants will take part in this study at approximately 15 to 20 study hospitals in the United States. Approximately [redacted] patients will be enrolled at this site.

The study sedation period will be up to 54 hours and only as long as sedation is required. After 54 hours, the treating doctor will decide whether to continue or stop sedation. If sedation is continued, you will receive standard of care sedation as prescribed by your doctors. You will be monitored for up to 7 days or until you are discharged from the hospital, whichever happens first. After 1 month, data regarding your hospital course will

[redacted]

be collected from your medical records, or with a brief phone call if the required information is not otherwise available in your record. At approximately 3 and 6 months after end of study treatment, you will receive phone or video calls to ask about your ability to think clearly, your quality of life, and your mobility and function. They will ask you questions about what you remember from your time in the ICU.

6. What will happen if you decide to be in this research study?

There are 3 stages to this study: Screening, Study Treatment Period, and Follow-Up.

Screening

If you agree to participate in this study, study staff will check to see if there is any reason you should not be in the study. Study staff will review and collect information about your medical history, surgical history, present health, and any medications that you are currently taking. We will ask you or your family questions about your ability to think clearly, quality of life, and mobility and function. These questions will take about 5-10 minutes, and you or your family will not have to answer any questions that make you or them feel uncomfortable. A blood sample will be taken to determine baseline safety assessments. For females who could become pregnant, a pregnancy test will be taken if not previously done during your hospital stay. The study team will ask for your contact information (phone numbers, email, and address) so that we can reach you after you leave the hospital to ask about your recovery.

Study Treatment Period

You will be randomized (like a flip of a coin) to determine which study treatment you receive inhaled isoflurane (administered via the Sedaconda ACD-S device) or propofol (administered via IV infusion) for sedation. You will have a 60% chance to receive isoflurane and a 40% chance to receive propofol.

If you undergo any procedures outside the ICU, study drug will be stopped. If you return to the ICU before 42 hours from starting the study drug treatment, study drug may be resumed if your condition still warrants receiving sedation. If you return to ICU later than 42 hours from start of study drug, you will transition to routine medical care at the discretion of the treating physician.

The bedside clinical nurse, study doctor, and study staff will know the study treatment and the doses that you are given. If the study medication (either isoflurane or propofol) you receive does not have the desired effect, you may receive other approved standard of care medications to achieve the sedation level prescribed by the clinical team. The appropriate level of sedation to be targeted will be determined by your treating physician and clinical team, not study staff.

Because this is a research study, the study treatment will be given to you only during the study sedation period and not after the study is over. You will not receive isoflurane for ICU sedation after the study period, but your doctors can choose to prescribe propofol or another routinely used sedative provided through the hospital after the study sedation period.

Propofol: If you are assigned to receive propofol, then propofol will be given through an IV directly into your vein.

Isoflurane: If you are assigned to receive isoflurane, then isoflurane will be administered via the Sedaconda ACD-S device, which connects between the ventilator and the breathing tube.

Monitoring: While you are sedated with study medication, study staff will closely monitor you, your overall health, your vital signs and physical exam, and your ongoing medical care, including medications that you are receiving. Study staff will frequently evaluate your sedation level, comfort, breathing pattern, and assess for any confusion. Your sedation tubing may be covered to ensure these assessments are not influenced by knowledge of which study medication you are receiving.

Blood samples: Blood samples will be collected and analyzed by the hospital clinical laboratory for safety monitoring. When able, these samples will be drawn from your existing lines to minimize discomfort. The total

[redacted]

amount of blood taken will be approximately 60 mL (4 tablespoons). For comparison, a standard blood donation at a blood collection center is about 475 mL of blood (about 96 teaspoons/2 cups).

Study treatment duration: The study sedation period will last for up to 54 hours and only as long as sedation is required. After this time, the treating doctor will decide whether to continue or stop sedation. If sedation is continued, you will receive standard of care sedation as prescribed by your doctors. If you are ready to come off the ventilator before end of the study sedation period, study medication will be stopped, and the breathing tube will be removed. You will not be kept on a ventilator any longer than your treating doctors think is medically necessary.

<u>Follow-Up</u>

After the study medication stops, the study team will monitor how fast you wake up, assess your comfort level, evaluate for confusion, and review your overall health and hospital course. These assessments will occur while you are in the hospital. You will not be kept in the hospital any longer than your treating doctors think is medically necessary.

At approximately 1 month after the study treatment, our site study team may call you or your representative to ask about any medications you may be taking and about your current health status if the study team cannot find this information in your medical records.

At approximately 3 and 6 months after end of study treatment, study team members from [redacted] University will call you by phone or video to ask about your ability to think clearly, quality of life, mobility and function, and what you remember from your time in the ICU. They will ask for your consent to conduct the phone surveys at the start of the call. The phone or video call will take about 1 hour. We will need contact information for multiple contacts to help ensure that their efforts to contact you will be successful. The [redacted] study team will request contact information (such as cell number, home number, work number, address, email) from you and additional contacts of your choosing. Any contact information you or your family choose to share with us will remain confidential and will be stored in a password protected database. This contact information will be shared with the study team at [redacted] University through a secure database, so they can perform the follow-up phone/video calls.

There are no outpatient (clinic) appointments associated with this study.

7. What are the potential benefits of participating in this study?

You may or may not benefit as a result of your participation in this study. With either study treatment, additional close monitoring from the study team may help ensure sedation is carefully adjusted to within the range prescribed by the treating physician. Isoflurane may or may not decrease the need for opioids (pain medications) while on the ventilator. Participants receiving isoflurane may or may not wake up faster once sedation is stopped. Results from this study may benefit others in the future and help us identify best options for sedation in patients on the ventilator in the ICU.

8. What are the risks of participating in this study?

All treatments have risks and may cause side effects. These may happen to you from the study treatment. These effects could be mild or serious. In some cases, these effects might be long lasting, permanent, or life-threatening. It is possible some risks may not be known at this time.

The study medications will only be given while you are in the ICU and on continuous monitoring. You will be closely observed for side effects throughout the study. The study doctor or study staff may give you treatment to help reduce any side effects or stop the study treatment early. The side effects most likely to happen to you if you take part in this study are noted below.

[redacted]

Risks of Propofol given Intravenously:

Propofol has been shown to be safe and effective for ICU sedation in well controlled clinical trials and was approved for the sedation of ICU patients on the ventilator in 1993. Propofol for ICU sedation is considered part of the standard of care in the United States and around the world. The dose and administration of propofol used in this study follows FDA-approved dosing instructions.

Common side effects of propofol (which occur in more than 1 in 10 patients) include a decrease in blood pressure, heart rate, or breathing effort. Discomfort, itching, or a rash at the IV site may occur occasionally.

Rare but potentially serious side effects of propofol (which occur in far less than 1 in 100 patients) include:

- Allergic reactions
- Abnormal heart rhythm
- Pancreatitis (inflammation of the pancreas, which can cause pain and nausea)
- Propofol infusion syndrome: a rare but life-threatening condition characterized by too much acid in the blood, high potassium, muscle breakdown, and/or heart, kidney, and liver failure. This rare event is most commonly reported at higher doses and longer durations than will be used in this study.

Risks of Isoflurane given Inhaled with the Sedaconda ACD-S:

Isoflurane has been shown to be safe and effective for deep sedation (anesthesia) during surgery, and is routinely used for that purpose in the United States and around the world. Isoflurane administered via the Sedaconda ACD-S is approved for sedation of ventilator-dependent ICU patients in Europe but not yet in the United States. The dose of isoflurane administered for ICU sedation is generally lower than required during surgery, and so dose-dependent side effects may be less common and less severe.

Common side effects of isoflurane (which occur in more than 1 in 10 patients) include a decrease in blood pressure, heart rate, or breathing effort. Nausea may occur occasionally.

Rare but potentially serious side effects of isoflurane (which occur in far less than 1 in 100 patients) include:

- Allergic reactions
- Abnormal heart rhythm
- Increased liver enzymes which may be a sign of liver dysfunction

Very rare but potentially serious side effect of isoflurane (which occurs in far less than 1 in 10,000 patients) include:

- Malignant hyperthermia (high body temperature, rigid muscles, rapid heart rate)

One adult patient with pre-existing high pressure in the brain was observed to have a further increase in pressure with isoflurane. Preliminary results from a study in children identified one participant with pre-existing high pressure in the brain who had a further increase in pressure with isoflurane. Patients with severe head injury or suspected to have high pressures in the brain are not eligible for this study.

Common side effects of using the Sedaconda ACD-S device include a slight increase in the amount of carbon dioxide in the blood. Rare potential side effects of the Sedaconda ACD-S include a decrease in the amount of sedation received over time or increase in breathing circuit resistance if the device becomes clogged by moisture or sputum.

Risks from Other Study Procedures:

- **Blood Samples:** Possible adverse effects from drawing blood include faintness, inflammation of the vein, pain, bruising, or bleeding at the site of puncture. There is also a slight possibility of infection.
- **Telephone or Video Assessments**: The follow-up telephone or video call assessments of your thinking, quality of life, and physical function could be emotionally uncomfortable. If this occurs, you

[redacted]

may choose to end the call at any time and are not required to answer any questions you do not want to answer.

Other Unknown Risks:

Since the use of Isoflurane administered via the Sedaconda ACD-S is an investigational study treatment, there may be other risks that are unknown. All medications have the potential risk of an allergic reaction, which if not treated promptly, could become life-threatening. Symptoms of an allergic reaction could be trouble breathing, or swelling of the face, mouth, lips, gums, tongue, or neck. Other symptoms of an allergic reaction may include rash, hives, or blisters. In the event of an allergic reaction, you will be treated promptly by the staff in the ICU. You will not be allowed to participate if you have a known allergy to propofol or if you or a family member has had a severe reaction to anesthesia (such as malignant hyperthermia). It is important that you tell the study doctor about any adverse changes in your health as soon as they occur, whether or not you think they are caused by the study treatment.

Risk of Loss of Privacy:

Every reasonable step will be taken to protect your privacy and confidentiality. Participation in any research study, including this one, may involve a risk of loss of privacy, and absolute confidentiality cannot be guaranteed. To minimize this risk, we will assign codes instead of using names and personal information. All information collected on paper will be kept in a secure location. Information collected on computer will be password protected and stored on a secure network. Staff at the study site will handle your personal information very carefully. We are required to make sure that people not involved with the study do not have access to your records. When results of the research are published or discussed at conferences, no information will be included that would reveal your identity.

9. What happens if there is new information?

Sometimes during the course of a research study, new information becomes available about the study treatment. The study doctor will inform you in a timely manner about any new important information that is discovered while you are in the study and discuss with you if you want to continue in the study. If this occurs, you may be asked to sign and date an updated consent form to confirm you agree to continue in the research study.

The study doctor may remove you from the study at any time without your consent if:

- Your study doctor does not consider it to be in your best interest to continue.
- Your study doctor has received new information about the safety or effectiveness of the study treatment that would cause you to no longer be able to participate.
- You cannot tolerate the study treatment.
- The study is stopped by the study site, the Sponsor, or regulatory authorities.
- For administrative reasons.

10. What happens if you are injured during the study?

If you are hurt or suffer other physical injury as a direct result of taking part in the study, the Sponsor will pay for the reasonable costs of medical treatment in accordance with applicable laws. The study site will treat your injury right away. The Sponsor has insurance to cover such costs, and will make these payments where the adverse effect or other physical injury resulted from:

- A medicine being tested or administered as part of the study, or
- Any test or procedure you received as part of the study.

The Sponsor will only pay for the medical costs that are not covered by your insurance or other programs. If you have medical insurance, check with your insurance company that taking part in this study will not affect your policy. There are no plans for the Sponsor to pay for any injury caused by the usual care you would

[redacted]

normally receive for treating your illness or the costs of any additional care. There are no plans for the Sponsor or study site to give you money for the injury. By signing and dating this document, you will not lose any of your legal rights or release anyone involved in the research from responsibility for mistakes.

11. What are the costs to you of taking part in the study?

There are no costs for you if you take part in this study. You will receive the study treatment at no charge, and you will not be charged for any study-related procedures. You are still responsible for paying for the usual care you would normally receive for the treatment of your illness. You, your insurance company, or some other third-party payer must pay for all other medicines and hospital costs.

12. Will you be paid to be in this study?

If you are enrolled and randomized into the study, you will receive a $50.00 check if you complete the 3-month follow-up phone/video assessment and a $50.00 check if you complete the 6-month follow-up phone/video assessment. [redacted] University is responsible for providing these payments to you, and they anticipate reimbursement checks should arrive in about 6-8 weeks after each follow-up phone/video call.

13. How will your privacy be protected?

Confidentiality

Once this consent form is signed and dated, you will be assigned a study code. During the study, the study doctor and study staff will collect information about you, including demographics, health data, and results of study procedures. Your records and study data (information) will not include your name or personal identity but will identify you with a study code. This code can only be tracked back to you via a code key that is held by authorized study personnel at the site. Although procedures are in place to protect your privacy, absolute confidentiality cannot be guaranteed.

Your participation in this study will be documented in your electronic medical record. This record can be viewed by authorized personnel from [redacted] and affiliated institutions because they share a medical record system. Study monitors and others who oversee the study may also need access to this record.

Authorization to Use and Disclose Protected Health Information

Access to your health information is required for this study. If you choose to take part in this study, you are giving us permission to use the protected health information and information collected during research that can identify you. The health information that we may collect and use for this research includes your past, present, and future physical or mental health and condition, and results of lab tests, examinations, or procedures. Information needed for this research may be obtained from any hospital, doctor, or other healthcare provider involved in your care. The research information that is shared with people outside of the study site, with the exception of research team members from [redacted] University who will be performing the follow-up calls, will not include your name, address, telephone number, or other direct identifiers unless disclosure of the information is required by law or you have authorized the disclosure. Study personnel are required by law to protect your health information.

By signing and dating this document, you authorize the study site to use and/or disclose (release) your health information for this research. Those who receive your health information may not be required by federal privacy laws to protect it and may share your information with others without your permission, if allowed by laws governing them. Your authorization to use and share your health information does not have an expiration (ending) date. In California and any other state that requires an expiration date, this authorization will expire 50 years after you sign this authorization document. You may change your mind and revoke (take back) this authorization at any time and for any reason. To revoke this authorization, you must write to the study doctor identified on the first page of this document. If you revoke your authorization, you will not be allowed to

continue taking part in the research. Also, even if you revoke this authorization, the Researchers and the Sponsor may continue to use and disclose the information they have already collected.

Data will be stored, processed, and compiled by the Sponsor both manually and electronically. Your collected information will be used and disclosed only in accordance with the law. Your identity will not be shared in any reports or publications resulting from this study. Your information will be identified only by your study code when sent to the Sponsor. Your study information may be disclosed to and used by:

- The study doctor and study staff
- Study team members at [redacted] University, who will be conducting the follow-up calls
- Sedana Medical AB, the study Sponsor paying for this research study
- Medpace, the contract research organization facilitating the study on behalf of Sponsor
- Authorized representatives and contractors of the Sponsor or Medpace
- U.S. Food and Drug Administration (FDA)
- Other agencies in the U.S. and other countries that have the authority to review study records
- Authorities from [redacted] University and [redacted] Hospital, including the Institutional Review Board (IRB)
- The Office of Human Research Protections (OHRP)
- Any successors to any of these organizations.

They are committed to protecting your privacy. As the research staff at the study site, we are required to make sure that people not involved with this study cannot see your research and medical information. We will keep your research files in a safe place and will handle your personal information very carefully.

A description of this clinical trial will be available on http://www.ClinicalTrials.gov, as required by U.S. Law. This Web site will not include information that can identify you. At most, the Web site will include a summary of the results. You can search this Web site at any time.

14. Who should I call if I have questions?

You may call the study doctor, Dr. [redacted], at [redacted] if you have any questions or concerns about this research study. If you have any questions about your rights as a research participant, you may contact: Institutional Review [redacted]. An institutional review board is a committee organized to protect the rights and welfare of human subjects involved in research.

[redacted]

STATEMENT OF CONSENT AND HIPAA AUTHORIZATION

I have read or had explained to me the information in this consent form. I believe that I understand this information. Any questions I had were answered. By signing and dating this consent, I am stating that I want to join this study. I do not give up any of my legal rights by signing and dating this consent form. I will receive a copy of this signed and dated consent form.

Research Participant: Print Name	*Research Participant: Signature*	*Date & Time*

– OR –

If study participant lacks capacity to consent, consent is given by LAR below.

LEGALLY AUTHORIZED REPRESENTATIVE

If consent was provided by a legally authorized representative (LAR), complete below.

You are being asked as the legally authorized representative (LAR) to permit the participant to take part in the research. By signing and dating below, you indicate you agree that the person named above can take part in this study.

LAR: Print Name	*LAR Relationship to Participant*
LAR: Signature	*Date & Time*

STUDY STAFF OBTAINING CONSENT

Study Staff: Print Name	*Study Staff: Signature*	*Date & Time*

[redacted]
